# Secretoglobin 3A1 in activated muscle satellite cells contributes to myosin heavy chain IIX and IIB fiber differentiation

**DOI:** 10.1007/s00018-025-06045-5

**Published:** 2025-12-31

**Authors:** Shigetoshi Yokoyama, Taketomo Kido, Mitsuhiro Yoneda, Danielle A. Springer, Parirokh P. Awasthi, Raj Chari, Andrew D. Patterson, Shioko Kimura

**Affiliations:** 1 Cancer Innovation Laboratory, Center for Cancer Research, National Cancer Institute, National Institutes of Health Bethesda, Maryland, 20892 USA; 2 Department of Veterinary and Biomedical Sciences, Penn State University, University Park, PA 16802 USA; 3 Laboratory of Cell Growth and Differentiation, Institute for Quantitative Biosciences, University of Tokyo, Tokyo, 113-0032 Japan; 4 Department of Oncology, Nagasaki University Graduate School of Biomedical Sciences, Nagasaki, 852-8523 Japan; 5 Murine Phenotyping Core Facility, National Heart, Lung, and Blood Institute, National Institutes of Health Bethesda, Maryland, 20892 USA; 6 Laboratory Animal Sciences Program, Frederick National Laboratory for Cancer Research, Frederick, MD 21702 USA

**Keywords:** Muscle satellite cells, PAX7, SCGB3A1, Muscle regeneration, Myosin heavy chain, Notch signaling

## Abstract

Skeletal muscle has an innate ability to restore damaged muscle fibers by contributing specific progenitor cells, called muscle satellite cells. Here we show that secretoglobin (SCGB) 3A1, a tumor suppressor gene in various malignancies including rhabdomyosarcoma, is induced just after muscle injury and contributes to damaged muscle fiber regeneration. Lineage tracing of SCGB3A1 in mice show that SCGB3A1-positive cells highly express myosin heavy chain (MyHC)-IIX in damaged fiber area. *Scgb3a1*-null and *Pax7*^*CreERT2*^;*Scgb3a1*^*f/f*^ conditional-null mice exhibit defective IIX and IIB fiber regeneration, with a concomitant reduction in the expression of *Notch3*, a gene important for the maintenance of satellite cell self-renewal pools. Aged *Scgb3a1*-null mice show reduced size of muscle fibers and mass, resulting in compromised muscle performance as compared to the age-matched wild-type mice. This study reveals that SCGB3A1 is an unexpected novel molecule expressed in muscle satellite cells that contributes to fiber type specific muscle regeneration.

**Supplementary Information:**

The online version contains supplementary material available at 10.1007/s00018-025-06045-5.

## Introduction

Skeletal muscle has a repeated ability to regenerate during injury and mechanical stress from environmental stimulants [1–4]. The regeneration of skeletal muscle is largely dependent on the muscle resident stem cells, called muscle satellite cells, which are mononucleated cells present between the basal lamina and plasma membrane of muscle fibers in the quiescent state. Satellite cells express paired box 7 (PAX7) transcription factor which is evolutionarily conserved [3, 5]. Upon receiving muscle damage signals, these cells migrate, proliferate, and fuse with each other to make terminally differentiated new muscle fibers on the damaged sites [1, 4].

Research has demonstrated that PAX7 is critical for normal adult muscle satellite cell function [5]. PAX7-expressing (PAX7(+)) satellite cells, upon receiving an injury signal, re-enter cell cycle and promote the myogenic program, expressing the master muscle regulatory transcription factors, such as MYOD/MYOD1 (myogenic differentiation 1), MYF5 (myogenic factor 5), MYOG (myogenin) and MRF4 (muscle-specific regulatory factor 4) [4]. The physiological balance of PAX7(+) satellite cells between self-renewal and differentiation is important for muscle homeostasis. Decreased self-renewal causes depletion of the muscle stem cell population, while the loss of control in self-renewal results in tumorigenesis [3]. Several molecules were reported to maintain the satellite cells balance such as NOTCH (neurogenic locus notch homolog protein) [6–10], EZH2 (enhancer of zeste 2 polycomb repressive complex 2 subunit) [11], FOXO3 (forkhead box O3) [12], DNMT3A (DNA methyltransferase 3 alpha) [13], PTEN (phosphatase and tensin homolog) [14, 15], MEGF10 (multiple EGF like domains 10) [16], and NELF (negative elongation factor) [17]. Loss of satellite cells is also known to occur with aging [18]. Despite identification of several proteins as markers of satellite cells, the underlying molecular mechanisms controlling various cell states (for example, activation, quiescence) of satellite cells are still not fully understood [4, 18]. It was reported that satellite cells are a heterogeneous population composed of self-renewal stem cells and committed progenitors produced by asymmetric division [10]. Furthermore, recent high-resolution transcriptome analysis (single-nucleus-RNA sequencing) revealed that the nuclei on a single myofiber are transcriptionally highly heterogenous for expression of the gene encoding myosin heavy chain (MyHC) [19, 20]. However, the molecular basis controlling MyHC fiber type specification (slow type I, fast type IIA, IIB and IIX in mouse; slow type I, fast type IIA and IIX in human) remains unknown [21–23].

Secretoglobin (SCGB) 3A1 (also known as UGRP2 (uteroglobin related protein 2) and HIN-1 (high in normal-1)) is a secreted type of small protein with tumor suppressive function [24–26]. We previously reported that SCGB3A1 protein is abundantly and specifically expressed in the lung and trachea, in both humans and mice [27, 28]. Specific expression of SCGB3A1 in lung airway epithelial cells may imply an important function for SCGB3A1 in the maintenance of lung homeostasis such as clearance of pathogens, in addition to tumor suppressor function, however, its role in other tissues, if any, has not been explored.

It was previously reported that aberrant methylation of the *SCGB3A1* promoter is a frequent event in many human malignancies. One such example is rhabdomyosarcoma (RMS, 61% frequency) [24], the most common soft-tissue sarcomas in children [29], exhibiting skeletal muscle features with aberrant myogenic differentiation. Although the expression of *Scgb3a1* was not confirmed in mouse adult skeletal muscle tissues [28], systematic RNAi screening of mouse cytokine genes in the mouse muscle myoblast cell line C2C12 revealed that knockdown for *Scgb3a1* impaired myoblast fusion, suggesting a crucial function for SCGB3A1 in muscle terminal differentiation. Further, it was also reported that *Scgb3a1* expression was not detected in C2C12 culture [30].

Based on these previous findings, we hypothesized that *Scgb3a1* is transiently expressed in a limited number of skeletal muscle cells (most likely in satellite cells) and plays a role in the maintenance of homeostasis. To address this hypothesis, we generated a knockout mouse for *Scgb3a1* (*Scgb3a1*^*-/-*^) and a conditional knockout mouse for *Scgb3a1* (*Scgb3a1*^*f/f*^) in muscle satellite cells using the *Pax7*^*CreERT2*^ transgene (*Pax7*^*CreERT2*^;*Scgb3a1*^*f/f*^). We carried out experiments using methods such as a muscle degeneration and regeneration model with barium chloride (BaCl_2_), single muscle fiber culture, histology, wholemount in situ hybridization, C2C12 cell culture, and SCGB3A1(+) cell lineage tracing using *Scgb3a1*^*GFP*^ and *Scgb3a1*^*CreERT2*^;*mT/mG* mice. The results revealed that *Scgb3a1* is expressed ubiquitously in early embryonic stages including myotome and dermomyotome in somites, while in adult tissues, it is specifically expressed in activated satellite cells, with the latter contributing to MyHC-IIX and IIB fiber regeneration and regulation of *Notch3* expression, which was reported to maintain satellite cell self-renewal [6, 10]. Moreover, the abrogated *Notch3* expression was found in *Scgb3a1*^*-/-*^ muscle satellite cells, leading to reduced regenerative abilities towards repeated muscle injury or in aged muscle tissues. These results suggest that SCGB3A1 regulates NOTCH signaling to maintain their self-renewal and regeneration abilities in injured or aged adult muscle and specifies the type of MyHC fibers.

## Results

### Establishment of *Scgb3a1*^*−/−*^ mice

**Fig. 1:**
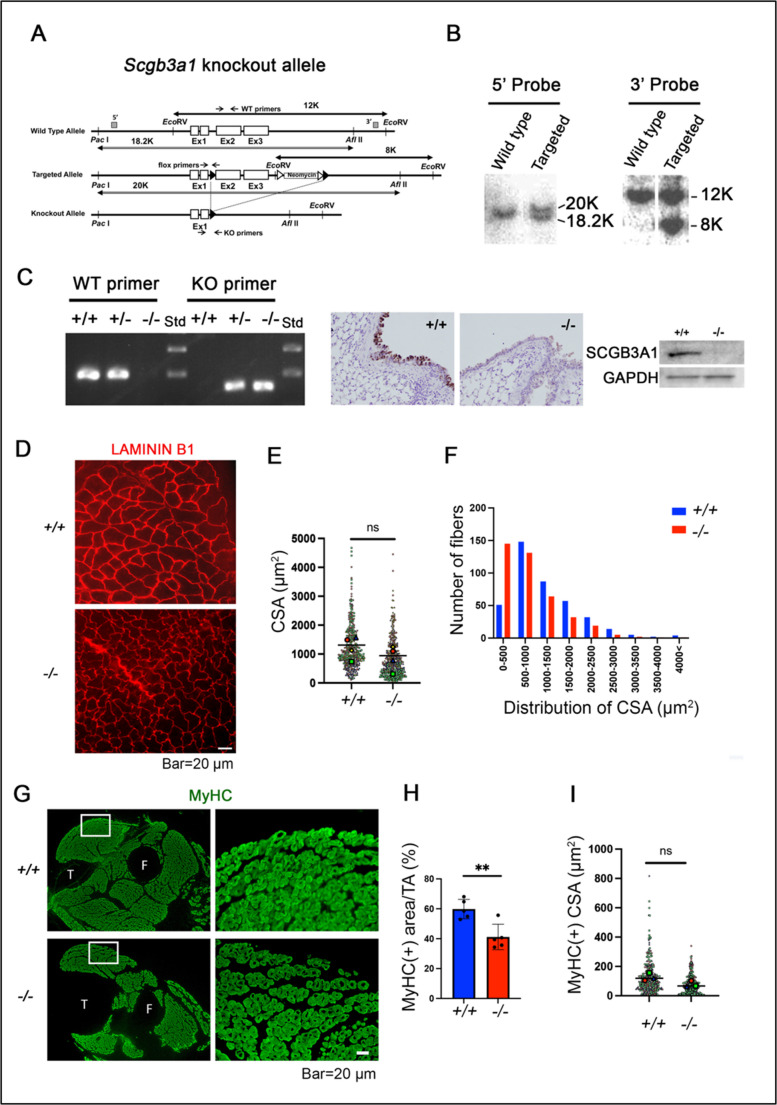
Establishment of *Scgb3a1* knockout mice. (**A**) Schematic illustration of the vector used to generate *Scgb3a1* knock-out mice. (**B**) Southern blotting results. The 5’ and 3’ probe was used to detect bands after digestion with PacI/AflII and EcoRV, respectively. The probe positions and the size of the band detected by each probe are shown in A. (**C**) (left) PCR genotyping. Wild-type (WT: +/+), 200 bp; knockout (KO: -/-), 163 bp. Std, molecular weight standard. Lower band, 200 bp; upper band, 300 bp. (right) SCGB3A1 protein expression by immunohistochemistry and western blotting using lung sections and tissues, respectively. (**D**) Immunostaining of LAMININ B1 in 4-week-old *Scgb3a1*^+/+^ and *Scgb3a1*^−/−^ TA muscles. Bar = 20 μm. (*n* = 3, representative image is shown). (**E** and **F**) Details of muscle fiber size distribution between *Scgb3a1*^+/+^ and *Scgb3a1*^−/−^ mice (*n* = 4 mice, each 100 fibers). (**G**) Immunostaining of MyHC for E17.5 hindlimb cross section of *Scgb3a1*^+/+^ and *Scgb3a1*^−/−^. right Fig. are magnified images of the white rectangle regions in the left images. T, tibia; F, fibula. Bar = 20 μm. (*n* = 3, representative image is shown). (**H**) Analysis of MyHC positive area excluding interstitial area within TA muscle (*n* = 5 independent fields). (**I**) Size of each MyHC (+) embryonic fibers (*n* = 3 mice, each 100 fibers). **p < 0.01 by Student’s *t*-test. ns, not significant

The *Scgb3a1* knockout mouse model (*Scgb3a1*^*−/−*^) was established on the C57BL/6NCr strain background (Fig. 1A-C). Four-week-old adult tibialis anterior (TA) muscle in *Scgb3a1*^*−/−*^ mice exhibited disorganized and variant size of muscle fibers (Fig. 1D). Especially, small size fibers (cross-sectional area: CSA < 500 µm^2^) were significantly increased in *Scgb3a1*^*−/−*^ (Fig. 1E, F), with no disability observed in the movement of adult *Scgb3a1*^*−/−*^ mice (cf. Fig. 5A).

Next, analysis of *Scgb3a1*^*−/−*^ mice for prenatal muscle defects using embryonic day (E) 17.5 hindlimb was performed (Fig. 1G-I). A reduced mass of embryonic MyHC (+) muscle fibers was observed in E17.5 *Scgb3a1*^*−/−*^ embryos (Fig. 1G, H), although the area of MyHC (+) CSA was not significantly different from those of *Scgb3a1*^*+/+*^ (Fig. 1I). To clarify if this size reduction of muscle simply reflects the developmental delay in *Scgb3a1*^*−/*−^, eighteen-week-old adult TA muscle was further analyzed, at the time when muscle development is generally considered complete (Supplementary Fig. S1). The results were similar to four-week-old muscles, suggesting that the size reduction is not due to the developmental delay in *Scgb3a1*^*−/−*^. These results suggest that *Scgb3a1* plays a role in the establishment of muscle mass from embryonic stages despite the fact that no obvious disability is found in the movement of adult *Scgb3a1*^*−/−*^ mice.

### Loss of *Scgb3a1* results in the impairment of muscle regeneration

*Scgb3a1*^*−/−*^ mice are grossly normal and fertile, with no obvious defects as revealed by gross physical movements. The expression of two major MyHC isoforms, MyHC-IIX and MyHC-IIB, was analyzed in intact TA muscles of adult *Scgb3a1*^*−/−*^mice (Supplementary Fig. S2). As a result, the expression levels of these functional MyHC isoforms were slightly reduced in *Scgb3a1*^*−/−*^TA muscles, although the decrease in MyHC-IIX was not statistically significant (Supplementary Fig. S2B-2D).

**Fig. 2:**
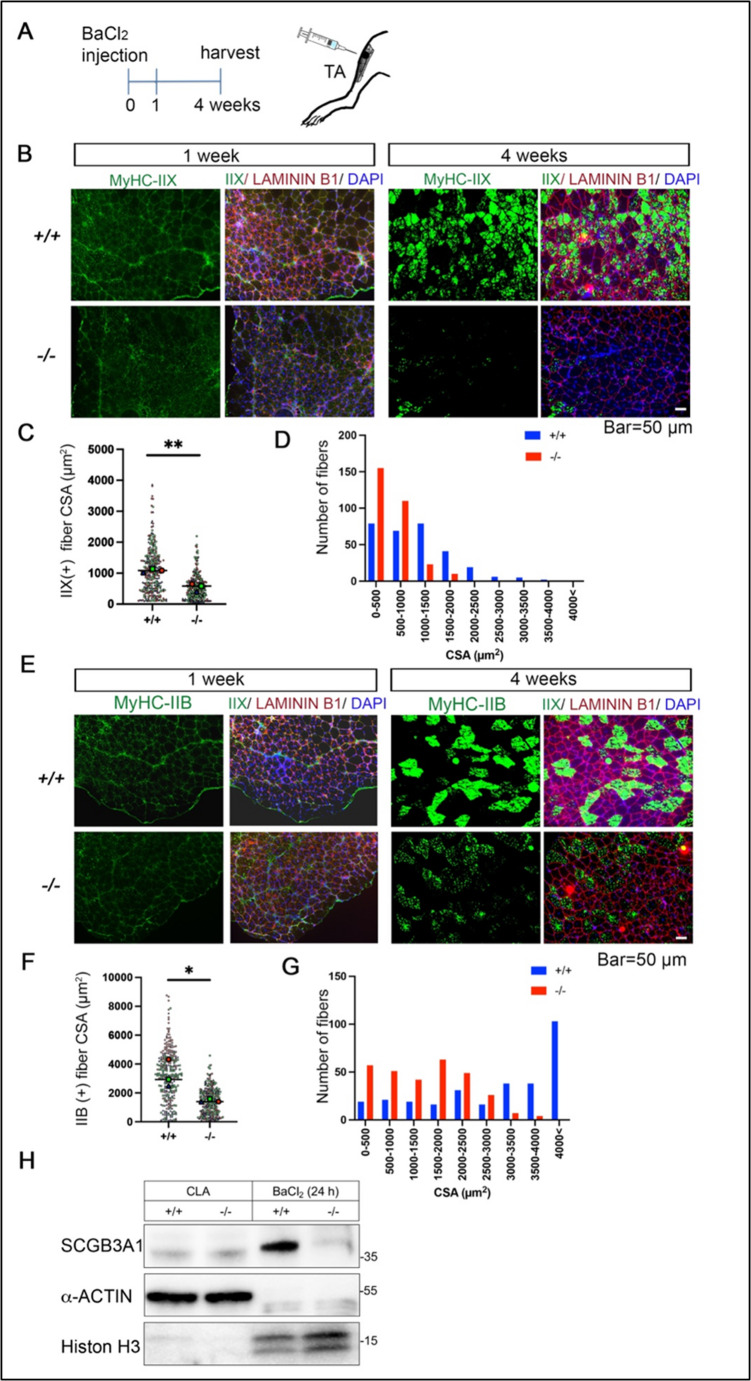
Muscle injury by injecting BaCl_2_ causes regeneration defects in *Scgb3a1*^*−/−*^. (**A**) Schematic illustration of BaCl_2_ injection and sample collection. BaCl_2_ injection was carried out at 3–4 months old. (**B**) Representative immunostaining images of MyHC-IIX and LAMININ B1 in TA muscle of *Scgb3a1*^*+/+*^ or *Scgb3a1*^*−/−*^ after one week and one month of BaCl_2_ injection. Nuclei were counterstained with DAPI. Bar = 50 μm. (n = 3) (**C**) IIX (+) fiber size distribution between Scgb3a1^+/+^ and *Scgb3a1*^*−/−*^ TA muscle after 1 month of BaCl_2_ injection (n = 3 mice, each 100 fibers). **(D**) Number of IIX fibers distribution after 1 month of BaCl_2_ injection shown in (C). (**E**) Representative immunostaining images of MyHC-IIB and LAMININ B1 in TA muscle of Scgb3a1^+/+^ or Scgb3a1^−/−^ after one week and one month of BaCl_2_ injection. Nuclei were counterstained with DAPI. Bar = 50 μm. (n = 3) (**F**) IIB (+) fiber size distribution between *Scgb3a1*^*+/+*^ or *Scgb3a1*^*−/−*^ TA muscle after 1 month of BaCl_2_ injection (n = 3 mice, each 100 fibers). (**G**) Number of IIB fibers distribution after 1 month of BaCl_2_ injection shown in (F). (**H**) Western blotting analysis for SCGB3A1 using whole cell extracts obtained from intact contra-lateral (CLA) TA and 24 h after BaCl_2_ injected injured TA muscle with α-ACTIN and Histon H3 antibodies respectively used for loading control. *p < 0.05, **p < 0.01 by Student’s t-test

Next, the regeneration abilities of skeletal muscle in *Scgb3a1*^*−/−*^ mice were analyzed by injecting BaCl_2_ solution into the hind leg TA muscle of 3- to 4-months-old mice (Fig. 2A). At day 7 after injury, while expression of MyHCs was barely found in either *Scgb3a1*^*+/+*^ or *Scgb3a1*^*−/−*^ mouse TA muscles (Fig. 2B, E), H&E staining revealed ineffective fiber recovery in *Scgb3a1*^*−/−*^TA muscles as characterized by large interstitial regions compared with control TA muscles (Supplementary Fig. S3, red arrowheads). On the other hand, embryonic MyHC (MyHC-emb), which is known to be re-expressed during early muscle regeneration and in many muscle diseases such as Duchenne muscular dystrophy [31], was more broadly detected in *Scgb3a1*^*−/−*^ TA muscles than in *Scgb3a1*^*+/+*^ muscles (Supplementary Fig. S3, white arrow).

After one month of regeneration, though TA muscle of *Scgb3a1*^*+/+*^ mice exhibited well differentiated adult fast MyHC (MyHC-IIX and -IIB)(+) fibers, *Scgb3a1*^*−/−*^ showed reduced adult MyHC expression levels (Fig. 2B-G). In contrast, the number of MyHC-IIA-positive fibers was increased in *Scgb3a1*^*−/−*^TA muscles after one month of regeneration, while the actual fiber size showed no significant difference (Supplementary Fig. S4). Slow MyHC (MyHC-I) was not expressed at sufficient levels to be detected in regenerated TA muscles (data not shown). These results suggest that *Scgb3a1*^*−/−*^mice exhibit impaired terminal differentiation of MyHC-IIX and MyHC-IIB fibers, accompanied by a compensatory upregulation of MyHC-IIA fibers.

In order to understand the precise timing of SCGB3A1 expression during muscle regeneration, western blotting was carried out. The results using anti-SCGB3A1 specific antibody suggest that the expression of SCGB3A1 was strongly induced just after injury in crushed muscle tissues by BaCl_2_ injection (Fig. 2H). These results suggest that SCGB3A1 is induced by muscle injury and can promote the regeneration of injured skeletal muscle, especially of type IIX and type IIB adult fast myosin heavy chains, while less effects on the regeneration of IIA type of MyHC.

### TA muscle of satellite cells-specific conditional knockout of *Scgb3a1* shows defects in regeneration

**Fig. 3:**
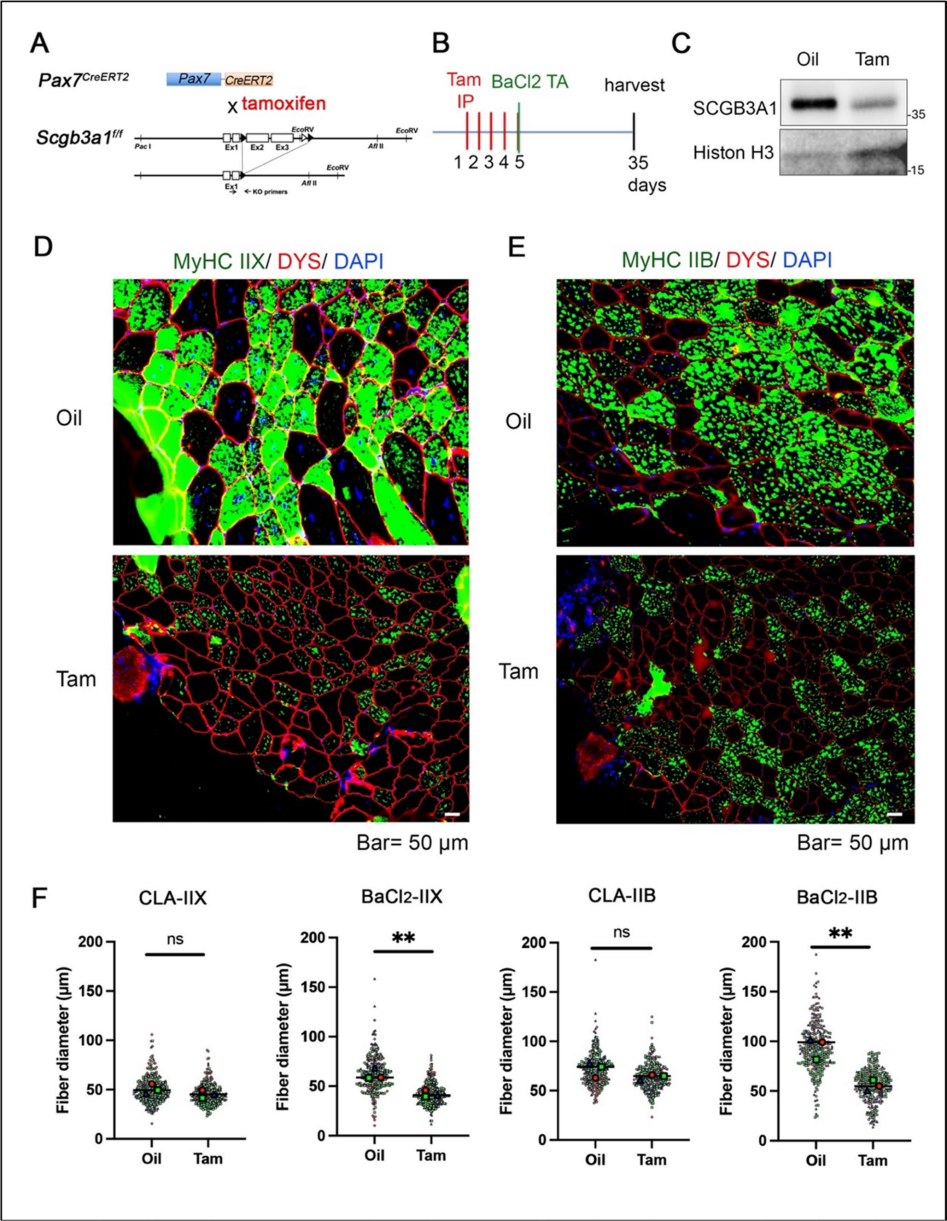
*Pax7*^*CreERT2*^; *Scgb3a1*^*f/f*^mice show reduced regeneration of BaCl_2_-induced injured muscle. (**A**) Schematic illustration of *Pax7*^*CreERT2*^*;Scgb3a1*^*f/f *^line. (**B**) Scheme for tamoxifen injection, BaCl_2_ injection and harvest of mice. Tamoxifen injection (Tam) was carried out at 4 weeks of age. (**C**) Western blotting analysis for the confirmation of Tam effects on SCGB3A1 deletion in BaCl_2_-injected TA muscles. (**D**,**E**) Representative immunostaining images of MyHC-IIX and DYSTROPHIN (**D**) or MyHC-IIB and DYSTROPHIN (**E**) in TA muscles of *Pax7*^*CreERT2*^*;Scgb3a1*^*f/f*^with (Tam) and without tamoxifen (Oil), obtained after 1 month of BaCl_2_ injection. Nuclei were counterstained with DAPI. Bar=50 µm. (n=3) (**F**) Comparison of MyHC-IIX (+) and IIB (+) muscle fibers diameter in mice with (Tam) and without tamoxifen (Oil), carried out after 1 month of BaCl_2_ injection. n=3 mice. 100 myofibers per mouse. CLA, contra lateral TA, ** *p*<0.01 by Student’s* t*-test. ns, not significant

In order to examine whether the phenotypes seen in *Scgb3a1*^*−/−*^ occur specifically in the muscle satellite cell lineage and whether *Scgb3a1* is indispensable for skeletal muscle regeneration in adult life, a tamoxifen inducible *Pax7*^*CreERT2*^;*Scgb3a1* ^*f/f*^ mouse model was established (Fig. 3A). Without tamoxifen, adult *Pax7*^*CreERT2*^;*Scgb3a1* ^*f/f*^ mice had normal TA muscle morphology and showed indistinguishable movement from *Scgb3a1*^*+/+*^ mice (data not shown). The *Pax7*^*CreERT2*^;*Scgb3a1* ^*f/f*^ mice received tamoxifen for five consecutive days to specifically delete the *Scgb3a1* gene in muscle satellite cells prior to BaCl_2_ injection into the TA muscle (Fig. 3B). Western blotting analysis confirmed the muscle injury-specific upregulation of SCGB3A1 protein in oil-injected control mice while it was significantly compromised by tamoxifen injection (Fig. 3C). MyHC expression was first evaluated in 4-weeks-old TA muscle of *Pax7*^*CreERT2*^;*Scgb3a1* ^*f/f*^ mice (conditional knockout, CKO) with or without tamoxifen without BaCl₂ treatment (Supplementary Fig. S5). The expression levels of MyHC-IIX and MyHC-IIB in CKO mice treated with tamoxifen were comparable to those treated with corn oil (control). These results suggest that, unlike in the conventional KO, deletion of *Scgb3a1* in the *Pax7* lineage after terminal muscle differentiation for four weeks is not sufficient to alter MyHC expression.

After 1 month of BaCl_2_ injection, well differentiated MyHC-IIX and IIB positive muscle fibers were found in oil-injected control *Pax7*^*CreERT2*^;*Scgb3a1*^*f/f *^mice. In contrast, few MyHC-IIX and IIB fibers were well differentiated in TA muscles of tamoxifen-injected *Pax7*^*CreERT2*^;*Scgb3a1*^*f/f *^mice (Fig. 3D, E). Statistically significantly reduced size of MyHC-IIX and II-B fiber diameters (Fig. 3F) and fiber area (Supplementary Fig. S6A-6 C) were found after tamoxifen injection in BaCl_2_-injeted *Pax7*^*CreERT2*^;*Scgb3a1*^*f/f*^ mice. On the contrary, the increased number of MyHC-IIA expression was detected in BaCl_2_-injected *Pax7*^*CreERT2*^;*Scgb3a1*^*f/f*^ mice (Supplementary Fig. S6D and 6E), although the fiber size was not significantly different (Supplementary Fig. S6F). These results are in good agreement with those observed in *Scgb3a1*^*−/−*^ muscles (cf. Figure 2). Of note, TA muscle hypertrophy occurred in control mice after BaCl_2_ injection, while it was not seen in tamoxifen-injected *Pax7*^*CreERT2*^;*Scgb3a1*^*f/f*^ mice (Fig. 3F). The results suggest that *Scgb3a1* is expressed in *Pax7*-positive satellite cells and the loss of SCGB3A1 results in the severe regeneration defects of MyHC-IIX and IIB fibers in adult TA muscle after injury.

### *Scgb3a1* lineage cells contribute to type IIX/IIB muscle regeneration

**Fig. 4:**
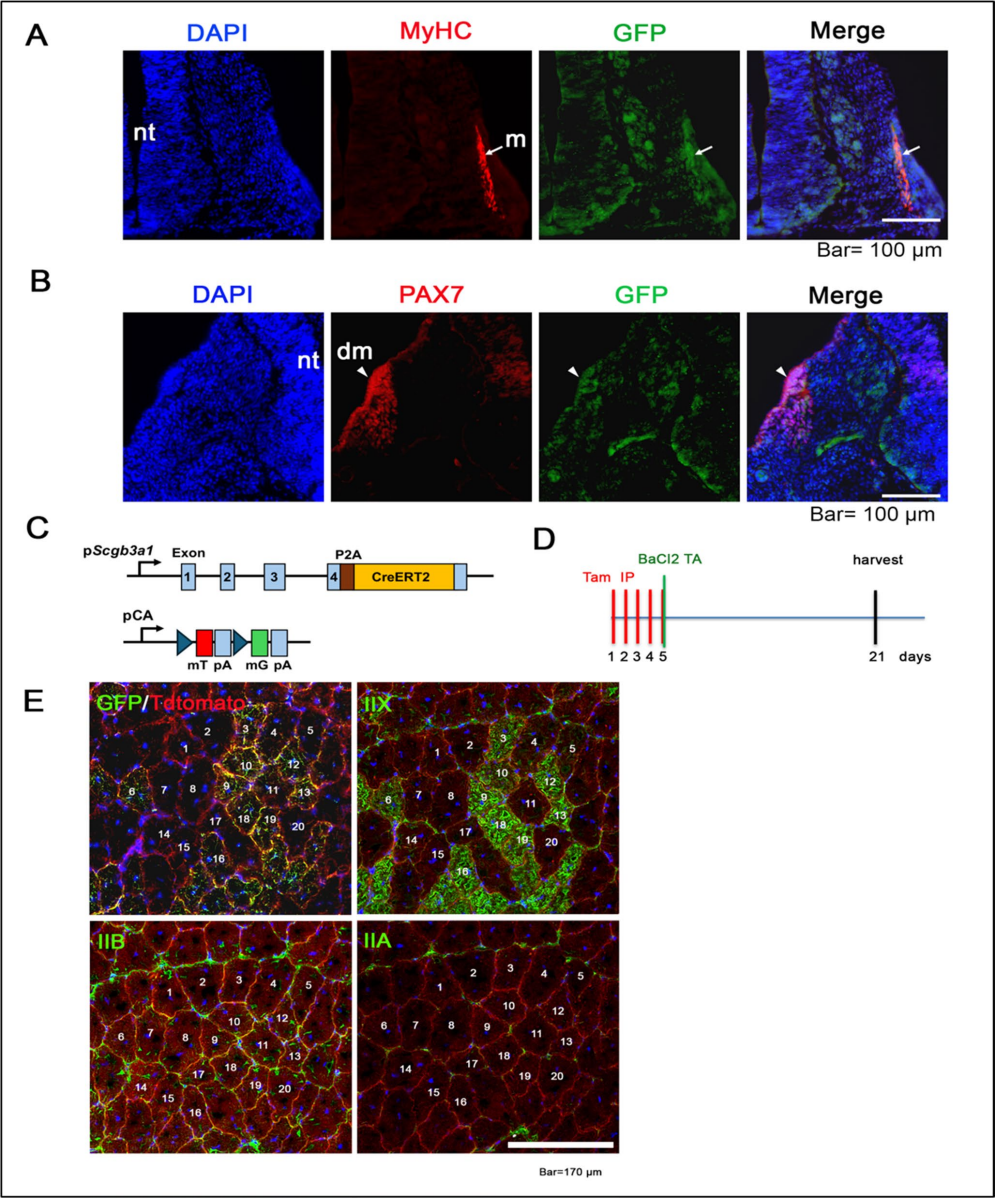
SCGB3A1(+) cells reside in embryonic somites and contribute to muscle fiber type specification during regeneration. (**A**) Immunofluorescent staining of SCGB3A1(GFP antibody) and MyHC (anti-MyHC antibody, clone MF20) of mouse E10.5 somite tissue. Arrows indicate the overlapped staining of MyHC and GFP in myotome (m). Nuclei were counterstained with DAPI. nt, neural tube. Bar = 100 μm. (**B**) Immunofluorescent staining of SCGB3A1(GFP) and PAX7 of mouse E10.5 somite tissue. Arrowheads indicate the overlapped staining of PAX7 and GFP in dermomyotome (dm). Nuclei were counterstained with DAPI. nt, neural tube. Bar = 100 μm. (C) Schematic illustration of *Scgb3a1*^*CreERT2*^;*mT/mG* line. (**D**) Scheme for tamoxifen injection, BaCl_2_ injection and harvest of mice. Tamoxifen injection was carried out at 4 weeks of age. (**E**) Double immunofluorescent staining of tdTomato (red) and GFP, MyHC-IIX, IIB, and IIA (green) in mouse TA muscle after three weeks of BaCl_2_ injection. Nuclei were counterstained with DAPI. Each number depicts same muscle fiber position in serial sections. Bar = 50 μm

Next, the lineage of *Scgb3a1*-expressing cells in early embryonic stages and after regeneration in adult muscle tissues was examined. These data would provide support for the reduced muscle regeneration ability of *Scgb3a1*^*−/−*^ and *Pax7*^*CreERT2*^;*Scgb3a1*^*f/f *^mice. For embryonic stages, *Scgb3a1*^*GFP*^ knock-in mice were established to analyze for the precise expression patterns of SCGB3A1 in embryonic somites (Fig. 4A, B). SCGB3A1-GFP was widely expressed in E10.5 mouse somite tissues and was co-localized with MyHC (myotome) and PAX7 (dermomyotome). These results suggest that SCGB3A1 contributes to primary myogenesis in early embryos. In situ hybridization was also performed using a DIG-labeled antisense RNA probe to determine the *Scgb3a1* mRNA distribution in early embryos (Supplementary Fig. S7A). *Scgb3a1* mRNA was faintly expressed in presomitic mesoderm (PSM) at E9.0 while it was relatively ubiquitously expressed in E11.0 (Supplementary Fig. S7A, B).

Next, to determine whether the SCGB3A1 cell lineage contributes to muscle regeneration in adult tissue, *Scgb3a1*^*CreERT2*^ knock-in mice were established and were crossed with *mT/mG*, double-fluorescent Cre reporter mice [32] (*Scgb3a1*^*CreERT2*^;*mT/mG*, Fig. 4C). These mice carry *tdTomato-EGFP* on *ROSA* allele and globally express tdTomato red fluorescence on the membrane, which changes to GFP in cells that express Cre in the presence of tamoxifen and all cells derived from this cell lineage. Three weeks after BaCl_2_ treatment (Fig. 4D), GFP (+) cells were found within regenerating muscle fibers which have a central nucleus (Fig. 4E, DAPI staining), and they were highly overlapping with the pattern of Type IIX fast MyHC fibers (Fig. 4E, Supplementary Fig. S8). Many of these GFP (+) IIX fibers were also faintly positive for IIB fibers (Fig. 4E, Supplementary Fig. S8C), suggesting the incompletely maturated IIX and IIB hybrid fibers. In further analysis of other samples, GFP (+) IIX (+) but IIB (-) or IIA (-) fibers were found among IIB (+) or IIA (+) fiber dominant region (Supplementary Fig. S8A, S8B, respectively). Considering that the mouse TA muscle is mainly composed of fast muscle fibers (with slow Type I fibers barely detectable [33]), it is most likely that the GFP (+) fibers are lineage of IIX or IIX/IIB hybrid fast muscle fibers. The results strongly demonstrate that SCGB3A1 is expressed in muscle precursor cells in early embryogenesis and their descendants contributing to the muscle fiber type specification and repairment in adult tissues after injury.

### Repeated BaCl_2_ treatments of *Scgb3a1*^*−/−*^ mice results in impairment on an inverted grip test

**Fig. 5:**
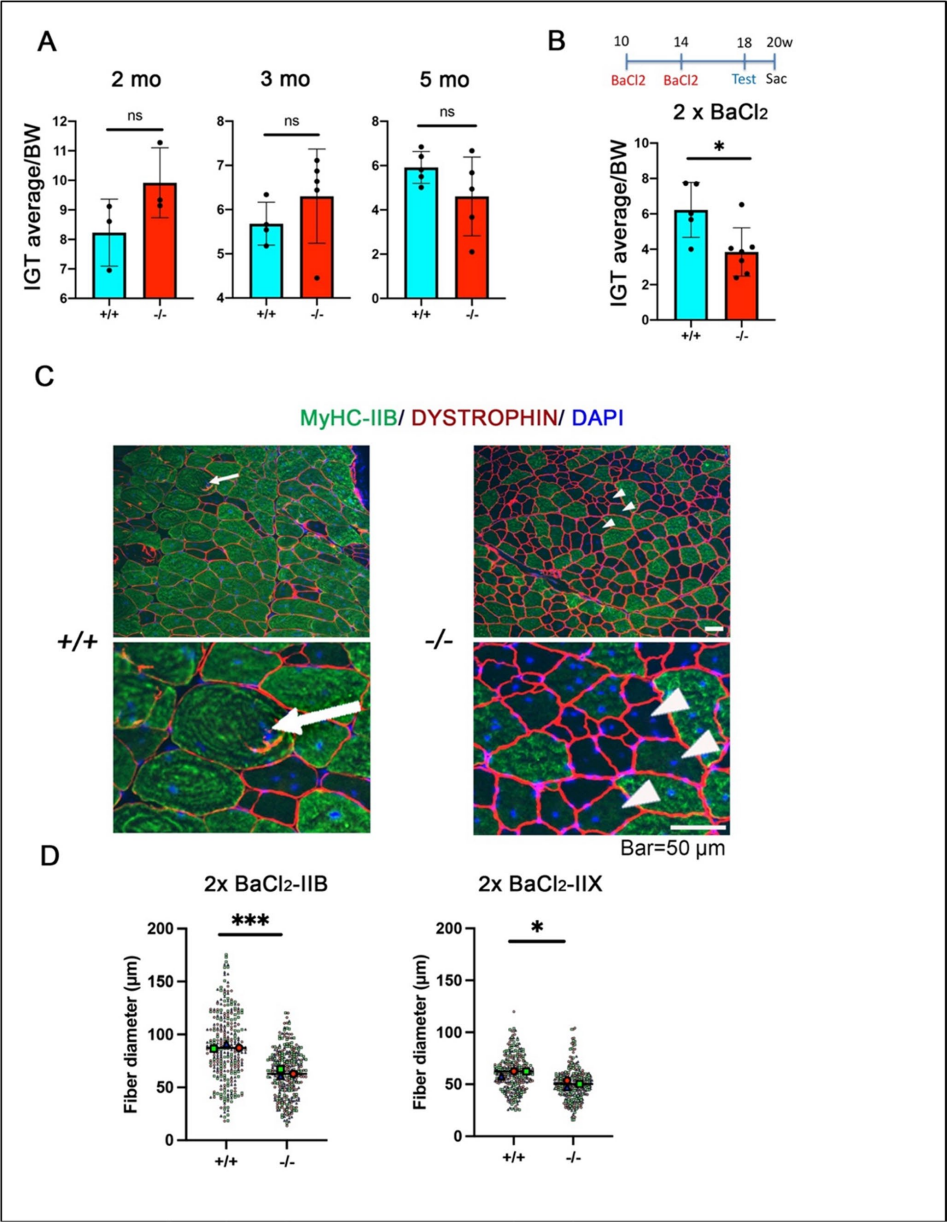
Regeneration defects after 2x BaCl_2_ injection affects the inverted grip test in *Scgb3a1*^*−/−*^ mice. (**A**, **B**) Inverted grip test (IGT) results of *Scgb3a1*^*+/+*^ and *Scgb3a1*^*−/−*^ mice. Duration time (in second) was normalized to each body weight (BW: gram). Test was carried out (**A**) using 2-month (n; +/+=3, -/-=3), 3-month (n; +/+=4, -/-=5), and 5-month (n; +/+=5, -/-=5)-old mice. (**B**) BaCl_2_ injection was given at 2.5 and 3.5 months of age, the test was carried out at 4.5 months of age, and the mice euthanized at 5 months of age (n; *Scgb3a1*^*+/+*^=5, *Scgb3a1*^*−/−*^=7). (**C**) Representative immunostaining images of MyHC-IIB and DYSTROPHIN in TA muscles after 2x BaCl_2_ injection. Arrow, representative muscle hypertrophy; arrowhead, reduced size MyHC-IIB-faintly positive or negative muscle fiber with central nucleus. Nuclei were counterstained with DAPI. Bar = 50 μm. (**D**) Average diameter size of each MyHC-IIB and IIX positive muscle fibers after 2x BaCl_2_ injection. *n* = 3 mice. 100 myofibers per mouse. *p < 0.05, *** *p* < 0.001 by Student’s *t*-test

To explore if loss of SCGB3A1 affects physical performance, *Scgb3a1*^*−/−*^ mice were subjected to twice BaCl_2_-induced injury model or no injury, followed by the inverted grid test (IGT). Without injury, *Scgb3a1*^*−/−*^ mice did not show any significant disabilities (Fig. 5A). However, repeated injury by BaCl_2_ injection caused shortening of the hanging time in the IGT test (Fig. 5B). Their muscle regeneration machinery was next examined by immunohistochemistry (Fig. 5C). In *Scgb3a1*^*+/+*^ mice, well organized hypertrophy of muscles fibers with strong MyHC-IIB positive staining was observed on TA muscle cross sections at six weeks after the 2nd BaCl_2_ injection (Fig. 5C arrow). In contrast, in *Scgb3a1*^*−/−*^ TA muscle, hypertrophy resulting from repeated BaCl_2_ injection followed by regeneration did not appear to occur and instead, many reduced size of faintly MyHC-IIB positive muscle fibers were seen (Fig. 5C, arrowheads). The reduction of fiber diameters after repeated BaCl_2_ injection was also seen in MyHC-IIX (Fig. 5D). These results demonstrate the critical function of *Scgb3a1* for muscle regeneration, especially following repeated injury.

### SCGB3A1 is expressed in PAX7(+) muscle satellite cells after BaCl_2_ injection

**Fig. 6:**
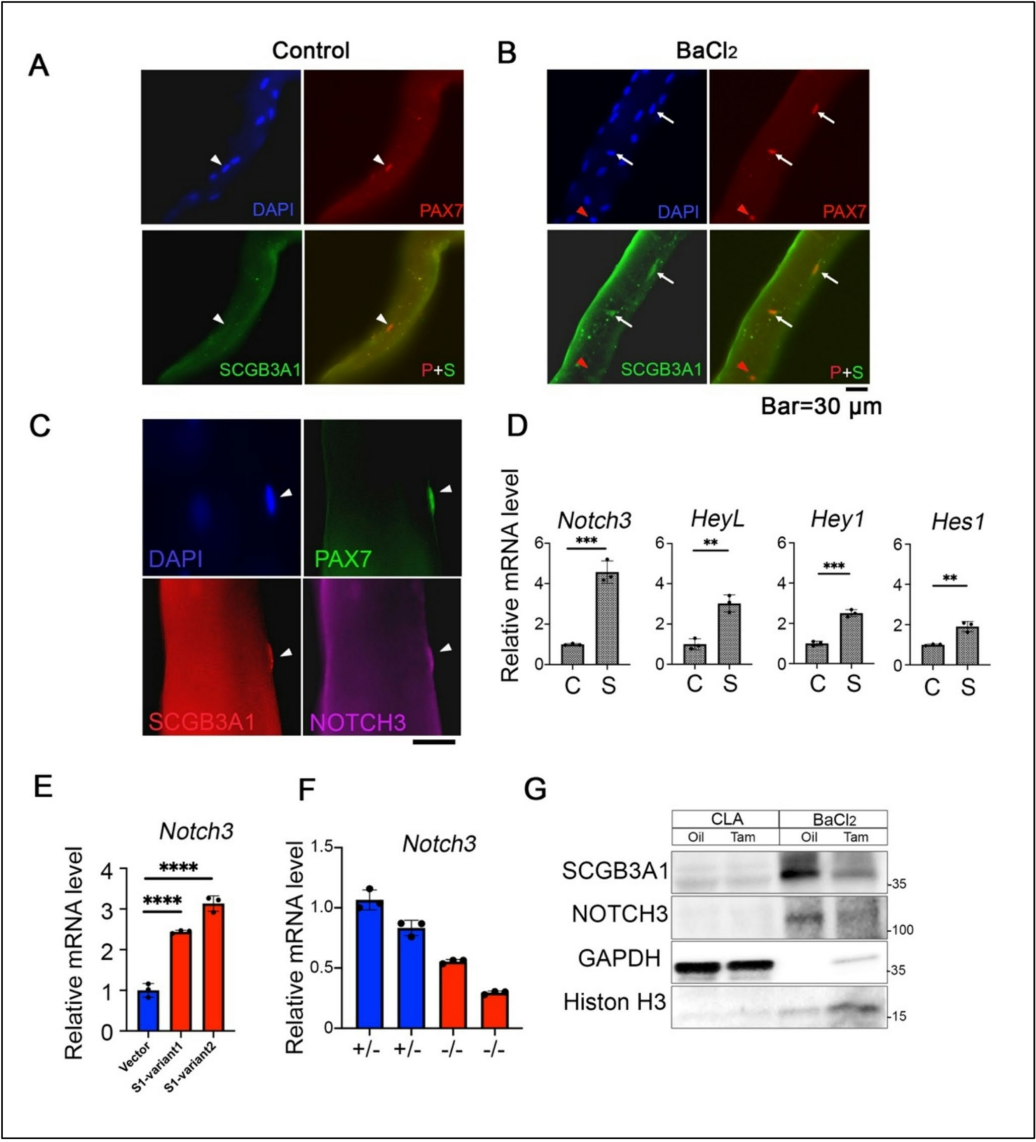
SCGB3A1 immunostaining co-localizes with PAX7 and NOTCH3 after BaCl_2_ injection. (**A**, **B**) Immunostaining of EDL muscle with (**B**) or without (**A**) BaCl_2_ injection carried out at 4 weeks of age. Muscles were collected 1 week after BaCl_2_ injection. Individually separated single muscle fibers were stained with anti-PAX7 and anti-SCGB3A1 antibodies. Arrowheads indicate PAX7(+)/SCGB3A1(-) satellite cells under control conditions (**A**). Arrows indicate PAX7(+)/SCGB3A1(+) and red arrowhead indicates PAX7(+)/SCGB3A1(-) cells after BaCl_2_ injection (**B**). (**C**) Immunostaining for PAX7 (green), NOTCH3 (purple) and SCGB3A1 (red) expression under BaCl_2_ injection. For all immunostaining, nuclei were counter-stained with DAPI. Bar = 30 μm. All data were representative images of three independent staining. (**D**) qPCR analysis and quantification of *Notch3*,* HeyL*,* Hey1*, and *Hes1* mRNAs with (S) or without (C) 10 ng/ml recombinant SCGB3A1. *n* = 3, in triplicate per sample. (**E**) *Notch3* expression in established cloned C2C12 cells overexpressing *Scgb3a1*. Both variant 1 and variant 2 sequences of *Scgb3a1* (S1-variant 1 and S1-variant 2, respectively, see Materials and Methods) increased *Notch3* mRNA in C2C12. (**F**) *Notch3* mRNA expression in E13.5 forelimb of the same litter of *Scgb3a1*^*+/−*^ or *Scgb3a1*^*−/−*^ embryos. *n* = 2, in triplicate per sample. (**G**) Western blotting analysis of CLA or BaCl_2_ injected TA muscle tissues of *Pax7*^*CreERT2*^;*Scgb3a1*^*f/f*^ mice for SCGB3A1 and NOTCH3 protein expressions. GAPDH and Histon H3 were used for loading control of CLA or injured TA muscle, respectively (*n* = 3). **p < 0.01, *** *p* < 0.001, **** *p* < 0.0001 by Student’s *t*-test

As described above, *Scgb3a1* deletion in both *Scgb3a1*^*−/−*^ and *Pax7*^*CreERT2*^;*Scgb3a1*^*f/f*^ resulted in the loss of muscle regeneration after injury. In order to determine the precise timing of SCGB3A1 expression in PAX7(+) satellite cells in adult skeletal muscles, single fiber immunostaining was performed using BaCl_2_-injected extensor digitorum longus (EDL) muscle (Fig. 6, A-C). While SCGB3A1 was hardly detectable in PAX7(+) cells in control fibers (Fig. 6A, white arrowhead), BaCl_2_-injected tissues showed some co-localization of PAX7 and SCGB3A1 (Fig. 6B, arrows). PAX7(+) satellite cells did not uniformly express SCGB3A1; some of these cells were negative for SCGB3A1 in BaCl_2_-treated fibers (Fig. 6B, red arrowhead). Approximately 70% of PAX7(+) cells were SCGB3A1(+) in BaCl₂-treated EDL muscle, which was more than twofold higher than under intact conditions (Supplementary Fig. S9A). We hypothesized that the heterogeneity of SCGB3A1 expression depends on the expression level of MyoD, which is induced in activated satellite cells. To confirm the status of SCGB3A1-positive satellite cells, triple staining with PAX7, MyoD, and SCGB3A1 antibodies was performed (Supplementary Fig. S9B). The results indicate that SCGB3A1 expression largely overlaps with MyoD, demonstrating that most SCGB3A1 is induced after satellite cell activation by injury.

The number of quiescent satellite cells (PAx7+/MyoD-) was next analyzed in control CKO EDL muscle (without BaCl_2_ treatment) four weeks after tamoxifen injection (Supplementary Fig. S9C). Tamoxifen administration reduced the number of PAX7+/MyoD- cells compared with oil-treated EDL muscle, while it increased the number of PAX7+/MyoD+ activated satellite cells, suggesting a failure to maintain the self-renewing satellite cell pool in CKO mice.

Furthermore, co-localization of SCGB3A1 with NOTCH3 (Fig. 6C) was seen in BaCl_2_ injury-induced muscle. NOTCH3 is reported to be expressed in either activated or quiescent satellite cells and maintain the number of self-renewal quiescent satellite cells [9]. Satellite cells with specific deletion of NOTCH signaling nuclear protein *RBP-Jk* resulted in the gradual disappearance of satellite cells [6]. Based on these reports and our results, we hypothesized that SCGB3A1 may have an important function for *Notch3* expression in muscle cells, thereby contributing to the maintenance of satellite cell quiescence.

### *Notch3* expression is regulated by SCGB3A1

In order to monitor the Notch downstream signaling in vitro, C2C12 myogenic cell lines were treated with or without recombinant SCGB3A2. *Notch3* and the target signaling genes (*HeyL* (hairy/enhancer-of-split related with YRPW motif-like protein), *Hey1* (hairy/enhancer-of-split related with YRPW motif protein 1), and *Hes1* (hairy and enhancer of split-1)) were significantly upregulated by SCGB3A1 addition to the culture medium (Fig. 6D). When a cDNA encoding SCGB3A1 was introduced into C2C12 using retrovirus, upregulation of *Notch3* was observed (Fig. 6E), but constitutive expression of *Scgb3a1* did not promote terminal differentiation (Supplementary Fig. S10,). Subsequently, reduced *Notch3* expression was observed in embryonic forelimb tissues in *Scgb3a1*^*−/−*^ mice (Fig. 6F). Furthermore, *Pax7*^*CreERT2*^;*Scgb3a1*^*f/f*^ TA muscle administrated Tam exhibited concomitant reduction of NOTCH3 protein expression along with SCGB3A1 (Fig. 6G). These results demonstrate a critical function for SCGB3A1 in the expressions of *Notch3* in myogenic cell lineage both in vivo and *in vitro.*

### Aged *Scgb3a1*^*−/−*^ mice exhibit skeletal muscle atrophy-like morphology

**Fig. 7:**
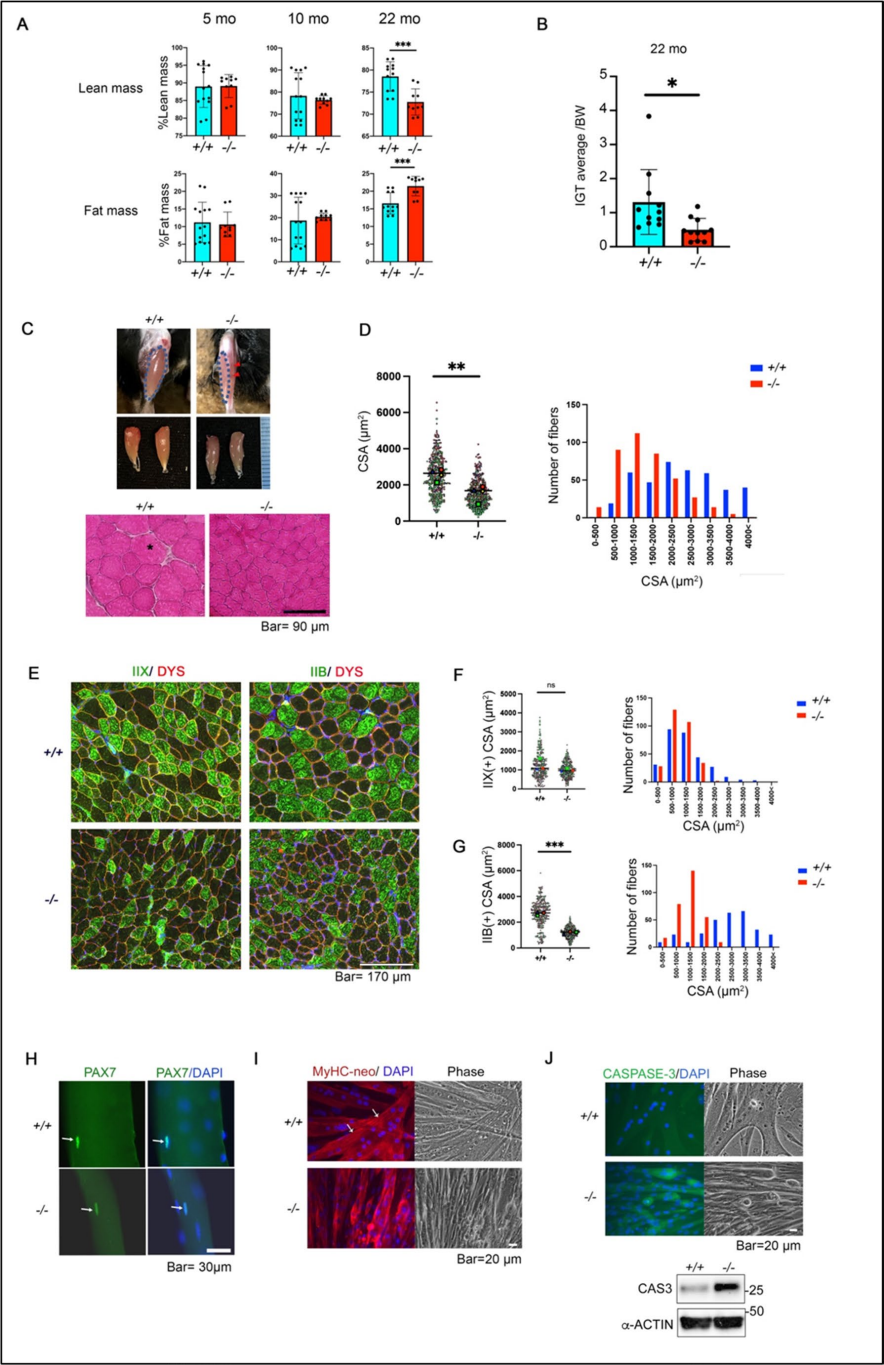
Aged *Scgb3a1*^*−/−*^ show reduced muscle mass and grip strength. (**A**) Comparison of body composition (lean mass and fat mass %) of *Scgb3a1*^*+/+*^ and *Scgb3a1*^*−/−*^ mice at various ages were compared. (**B)** Inverted grip test (IGT) results of *Scgb3a1*^*+/+*^ and *Scgb3a1*^*−/−*^ mice carried out at 22-month of age. A dot indicates a mouse. *p < 0.05, ***p < 0.001 by Student’s *t*-test. (**C**) Typical images of 22-month-old *Scgb3a1*^*+/+*^ and *Scgb3a1*^*−/−*^ mice TA muscle (depicted with dotted blue line). Red arrowheads indicate thinner TA muscle of *Scgb3a1*^*−/−*^ mice. (bottom) H&E staining of same specimen. Asterisk indicates the biggest muscle fiber in size in *Scgb3a1*^*+/+*^ TA muscle, while these big muscle fibers are rarely seen in *Scgb3a1*^*−/−*^ tissue. Bar = 90 μm. (**D**) Total TA muscle CSA (µm^2^) and its distribution (*n* = 4 mice, each 100 fiber per mouse). (**E**) Immunostaining of MyHC-IIX and IIB of 22-month-old TA muscle. Bar = 170 μm. (**F** and **G**) Total IIX (**F**) or IIB(**G**) (+) fiber area (µm^2^) and detailed size distribution of each MyHC fibers both in *Scgb3a1*^*+/+*^ and *Scgb3a1*^*−/−*^. *N* = 3 mice, each 100 fibers per mouse. ***p < 0.001 by Student’s *t*-test. ns, not significant. (**H**) Representative immunostaining of PAX7 of 22-month-old EDL single fibers (arrows). (**I**) Representative immunostaining of muscle satellite cells culture (P5) stained with anti-MyHC-neonatal antibody. White arrows indicate striated fibers in *Scgb3a1*^*+/+*^ satellite cells. Nuclei were counterstained with DAPI. Bar = 20 μm. (**J**) Caspase-3 immunostaining of muscle satellite cells culture (P5). Nuclei were counterstained with DAPI. Bar = 20 μm. (bottom) Western blotting analysis for caspase-3 (CAS3) expression in P5 *Scgb3a1*^*+/+*^ and *Scgb3a1*^*−/−*^ satellite cells cultures

Our findings thus far suggest that the regenerative abilities of skeletal muscle in *Scgb3a1*^*−/−*^ mice are reduced after repeated injury. Aged muscle is also known to have the reduced abilities in regeneration. To examine whether aged *Scgb3a1*^*−/−*^ mice have muscle mass reduction without injury, body composition analysis (fat mass % and lean mass %) of *Scgb3a1*^*−/−*^ mice was carried out using those from 5-months-old to 22-months-old (Fig. 7A). As expected, the body composition was different in 22-month-old aged mice, showing reduced muscle mass while increased fat percentage (Fig. 7A). In concordance with this, the IGT/BW ratio determined at 22 months of age was reduced for *Scgb3a1*^*−/−*^ mice as compared with *Scgb3a1*^*+/+*^ mice (Fig. 7B). The representative gross picture of the TA muscle of aged *Scgb3a1*^*+/+*^ and *Scgb3a1*^*−/−*^ mice at 22 months showed that *Scgb3a1*^*−/−*^ mice TA was thinner and presented a pale color as compared with *Scgb3a1*^*+/+*^ mice (Fig. 7C). Hematoxylin & Eosin (HE) staining of the TA sections showed that a fiber in *Scgb3a1*^*−/−*^ TA was not nearly as big as those seen in *Scgb3a1*^*+/+*^ TA (* is the biggest fiber in +/+ mouse, Fig. 7C, bottom), and the CSA sizes in *Scgb3a1*^*−/−*^ mice were severely reduced as compared to *Scgb3a1*^*+/+*^ mice (Fig. 7D). Immunostaining of MyHC-IIX and IIB confirmed the reduced muscle differentiation in the *Scgb3a1*^*−/−*^ mice, especially in IIB fibers (Fig. 7, E-G). Importantly, weakly scattered slow MyHC (MyHC-I) fibers were markedly increased in aged *Scgb3a1*^*−/−*^ TA muscle compared with WT mice, which represents a key fiber transition to hybrid type (type II to type II/I) frequently associated with sarcopenia—the age-related loss of muscle mass and function (Supplementary Fig. S11A, white arrowheads) [34–36]. Finally, the satellite cells derived from the single fiber of aged *Scgb3a1*^*−/−*^ were evaluated. Diameter of aged *Scgb3a1*^*−/−*^ EDL single fibers were significantly thinner than that of *Scgb3a1*^*+/+*^ (Fig. 7H; Table 1) and as expected, PAX7(+) satellite cells number was drastically reduced in aged EDL (Table 1). Importantly, we found that the proportion of quiescent satellite cells (PAX7+/MyoD-) significantly reduced in aged *Scgb3a1*^*−/−*^, supporting that SCGB3A1 may contribute to satellite cell self-renewal (Supplementary Fig. S11C). *Scgb3a1*^*−/−*^ satellite cells in culture started to show failure to make terminally differentiated myotubes at advanced passages (P5) in vitro (Fig. 7I). Caspase proteins are known to be involved in various types of cell death including progressive muscle fibers degeneration in Duchenne muscular dystrophy [37]. Caspase-3, the executer of apoptosis conducted by caspases signaling [38], was accumulated in *Scgb3a1*^*−/−*^ cells, indicating that these satellite cells were degenerating as the time in culture increased (Fig. 7J). These results support our conclusion that the myofibers of *Scgb3a1*^*−/−*^ mice exhibit characteristic features of impaired muscle satellite cell maintenance and terminal differentiation under aging and severe muscle injury, displaying sarcopenia-like phenotypes such as reduced grip strength, size reduction of type II fibers, muscle atrophy and fiber-type transition towards type I fibers.

**Table 1:** Number of PAX7(+) satellite cells and average diameter of muscle fiber in aged mouse

	+/+	-/-
PAX7 + nucleus (n)	32	18
Fibers (n)	17	20
Average PAX7 + satellite cells/fiber	1.9	0.9
Average fiber diameter (µm)	59.8	41.5

## Discussion

Skeletal muscle analysis, especially the process of satellite cell regeneration, is a good model to determine the mechanism, homeostasis, and regeneration of muscles. Notably, skeletal muscles have their specific transcriptional programs by sequentially expressing *Pax3/7* in progenitor cells, and *MyoD* family transcription factors for committed myogenic cells. For their activation, satellite cells interact with various molecules/signals derived from the extracellular matrix, immune cells, nerve, and blood vessels [18, 39]. Previous reports suggest that these satellite cell ‘niche’ also changes with age and both satellite cell intrinsic function and alternation of the niche impact the regenerative abilities of muscle tissues [40, 41].

NOTCH signaling has been extensively reported as a major regulator of satellite cells proliferation and self-renewal. Knockout of Notch ligand (*Delta1*) [42], or a nuclear mediator of Notch signaling (*RBP/J*) [43], results in similar deficits in embryonic myogenic cells and depletion of satellite cells. It was also reported that the enhancement of NOTCH signaling promotes muscle regeneration in aged muscle [7]. Others reported that inhibition of NOTCH signaling resulted in the loss of Pax7(+)/Myf5(-) self-renewal cells, suggesting that activation of the NOTCH signaling pathway plays a positive role in the self-renewal of satellite stem cells [10].

Our findings demonstrate that SCGB3A1 is a novel protein expressed in PAX7(+)-activated satellite cells. Knockout of SCGB3A1 causes defects in regeneration, specifically affecting differentiation of adult fast MyHC-IIX and IIB fibers (see discussion below) and age-related reduction of functional satellite cells, leading to downregulation of NOTCH3. In addition, the fact that SCGB3A1 is minimally detected in intact muscle fibers, while abundantly found in TA muscle after treatment with BaCl_2,_ strongly suggests that this protein is induced when muscle fiber damages occur.

Recent evidence suggests that satellite cells are a much more heterogeneous population of cells than previously thought, allowing them to rapidly adjust to the extrinsic environmental changes [39]. Muscle fiber types in mammals are also composed of heterogeneous fiber types; one slow twitch type (Type I) and three fast twitch types (Type IIA, IIB and IIX) in mice and two fast twitch fibers (IIA and IIX) in humans [22, 31]. Furthermore, they exist as hybrid fibers bridging the gaps between pure fiber types (cg. I ↔ I/IIA ↔ IIA ↔ IIA/IIX ↔ IIX ↔ IIX/IIB ↔ IIB) [33, 44]. While type I and IIA fibers exhibit oxidative metabolism, type IIB and IIX are glycolytic and are more susceptible to a variety of atrophic signals than type I fiber [22]. Using a lineage tracing method, Twist 2 (twist-related protein 2) (+) progenitors were reported to contribute specifically to the regeneration of type IIB and IIX myofibers in adult mice, suggesting the possibilities that there are fiber-specific progenitor cells which remains largely unexplored [21]. Moreover, it was shown that Twist 2 did not contribute to primary and secondary myogenesis as it was not expressed in early myotome [21]. This contrasts to our results with SCGB3A1. SCGB3A1 is expressed in the early dermomyotome and myotome colocalizing with PAX7 and MyHC, respectively, and *Scgb3a1*^*-/-*^ mice have prenatal muscle mass reduction.

Our lineage tracing results suggest that SCGB3A1 is mainly expressed in regenerating MyHC-IIX fibers. Considering the IIB fibers were also largely compromised by knockout of *Scgb3a1*, it is likely that SCGB3A1(+) IIX fibers also contribute to the differentiation of IIB fibers probably as IIX/IIB hybrid type which are bridging single IIX and IIB fibers. These results suggest that SCGB3A1 is expressed in PAX7(+) myogenic precursor cell lineage and has some function for prenatal myogenesis in addition to contributing to the specification of MyHC-IIX lineage in adult muscle fibers.

Considering that promoter hypermethylation of *SCGB3A1* is frequent in various cancer cells including RMS [24, 43], it is possible that *SCGB3A1* expression might also be epigenetically regulated in normal skeletal muscle differentiation events, including satellite cells self-renewal, fiber types specification, and regulation of the NOTCH signaling. Further studies are required to address these questions.

Of specific interest is a previously published study on the NOTCH3 interactome which showed that SCGB3A1 is one of the candidates among 40 proteins to interact with the NOTCH3 receptor [45]. Furthermore, we previously demonstrated that SCGB3A2 (also called UGRP1 (uteroglobin-related protein 1)), another member belonging to the same SCGB3A gene subfamily as SCGB3A1, is a ligand for Syndecan-1 (SDC1) in cancer cells [46, 47]. In muscle cells, the Syndecan family of receptors (SDC3/4) are known as a marker for quiescent satellite cells [48]. Importantly, SDC3 is required for the processing of NOTCH and cooperatively regulates satellite cells pool [49]. In the current study, *Scgb3a1* was broadly expressed from early embryogenesis and *Scgb3a1*^*-/-*^ mice exhibit reduced muscle differentiation and regeneration. Combining the previous literature, our new findings suggest that SCGB3A1 could be a self-ligand to interact with the NOTCH/SDC3 complex in satellite cells and may play a role in controlling self-renewal and differentiation of both embryonic myogenetic precursors and adult satellite cells, contributing to the specification of MyHC fiber types, and preventing muscle cells from tumorigenesis. Further analysis on the molecular intrinsic (cell-cell) and extrinsic (cell-niche) interactions of SCGB3A1 is critical for understanding the role of SCGB3A1 in muscle regeneration and tumorigenesis.

Muscle fibers are highly heterogenous population and complicated numerous factors including gene regulation and environmental cues, determine the specific fiber types. To our knowledge, few molecules are reported to contribute to specification of adult MyHC-IIX and IIB fibers. Both are anaerobic metabolism type and produce powerful contraction, and are essential for supporting aging body. Considering human only has IIX fibers, our finding that SCGB3A1-descendant cells highly overlap with MyHC-IIX–expressing fibers, could shed light on the clinical study of aging muscle fibers. Furthermore, high percentage of aberrant methylation of *SCGB3A1* promoter in rhabdomyosarcoma patients indicates SCGB3A1 should be considered as a potential clinical target for assessment of the most common type of soft tissue sarcoma for children.

## Materials and methods

### Antibodies and chemical reagents

All antibodies and chemical reagents used in this manuscript are summarized in Supplementary Table S1.

### Generation of ubiquitous and muscle satellite cell-specific *Scgb3a1*^*−/−*^ mice

Generation of *Scgb3a1*^*−/−*^ mice was carried out using homologous recombination of a targeting vector generated by recombineering, followed by embryonic stem (ES) cell injection (Fig. 1A). During production of *Scgb3a1*^*−/−*^ mice, *Scgb3a1*-conditional mice with the *Scgb3a1*-floxed allele (*Scgb3a1*^*f/f*^) were also generated. Recombineering was performed as described [50]. After successive recombineering procedures, the final targeting vector was purified and electroporated into C57BL/6J x 129/SvJae hybrid ES cells. G418-resistant ES cells were genotyped by Southern blotting using 5’, 3’ and Neo probes. Two correctly targeted ES clones were injected into blastocycts obtained from C57BL/6NCr mice. Germline transmission in offspring of *Scgb3a1*-targeted chimeric mice crossed with wild-type C57BL/6NCr was confirmed by Southern blotting and PCR analyses (Fig. 1B, C). They were crossed to EIIA-Cre transgenic mice (FVB background, Jackson Laboratory #003724) to remove the sequence flanked by the two loxP sites to generate a complete knockout allele (*Scgb3a1*^*−/*−^), while beta-actin promoter-driven Flp transgenic mice [51] were used to remove the Neo cassette to produce *Scgb3a1*-floxed allele (*Scgb3a1*^*f/f*^). Heterozygous mice with the *Scgb3a1* knockout allele were intracrossed to produce homozygous *Scgb3a1*^−/−^ mice while those with the floxed allele were crossed with *Pax7*^*CreERT2*^ allele (Jackson Laboratory, strain #017763) to produce homozygous conditional-knockout mice in satellite cells (*Pax7*^*CreER2T*^;*Scgb3a1*^*f/f*^). *Scgb3a1*^−/−^ mice were backcrossed at least six generations onto C57BL/6NCr inbred background. Wild-type (*Scgb3a2*^+/+^) and *Scgb3a1*^−/−^ mice were maintained under standard specific-pathogen-free conditions. The primers for detecting knockout allele were: KO-F: 5’-GGTGTTGCTTTCTTCATGGAC-3’, KO-R: 5’- AGCTCGGTGACACACTTCCT-3’, with a 163 bp product. The primers for detecting wild-type *Scgb3a1* gene with the sequences corresponding to a part of the exon 2 were: EX2-F: 5’-TGGCCACCTTTCCTAACTTC-3’, EX2-R: 5’- AAGGGGAAACTGGGTGACAT-3’, with a 200 bp product (Fig. 1A).

### Generation of *Scgb3a1*^*GFP*^ and *Scgb3a1*^*CreERT2*^ knock-in transgenic mice

Generation of *Scgb3a1* knock-in transgenic mice was carried out first by designing guide RNAs near the end of the protein coding sequence of mouse *Scgb3a1* using sgRNA Scorer 2.0 [52] (Supplementary Table S2). To assess activity in cultured cells, in vitro transcribed (IVT) guide RNAs and recombinant Cas9 protein were complexed and transfected into P19 cells. After 48–72 h, DNA was extracted using Quick Extract (Lucigen) and subjected to deep amplicon sequencing using the Illumina MiSeq [53]. Candidate 1537 (Supplementary Table S2) performed the best and subsequently, a chemically modified version of this guide RNA was obtained from Synthego. Homology directed repair template was generated in two steps. First, double stranded plasmid was assembled using synthesized DNA (Twist Biosciences), PCR fragments containing either G4S3-GFP (pCE0680) or P2A-Cre-ERT2 (pCE0681), and digested plasmid pRC0082 (Addgene# 195320) using isothermal assembly [54]. Second, single stranded DNA (ssDNA) was made using the Guide it Long ssDNA Production system (Takara) with PCR product generated from the respective plasmids (pCE0680, pCE0681) using the primers listed in Supplementary Table S2. Recombinant Cas9 protein, synthetic guide RNA, and single stranded donor DNA were then complexed and microinjected in fertilized eggs as previously described [55, 56].

### Southern blot analysis

Genomic DNA was isolated from tail biopsy and digested with Pac I and Afl II for 5’ probe or EcoRV for 3’ probe. Digested DNA fragments were run on a 0.4% agarose gel overnight and were transferred to a nylon membrane. A ^32^P-labeled (PerkinElmer) probes were prepared using the Ready-To-Go DNA Labeling Beads (-dCTP) (GE Healthcare Life Sciences). Signals were detected using Storm 840 (GE Healthcare Life Sciences).

### Animal studies

In order to induce Cre mediated *Scgb3a1* gene deletion in adult muscle satellite cells, *Pax*7^CreERT2^;*Scgb3a1*^*f/f*^mice were injected intraperitoneally with 50 mg/kg tamoxifen (Sigma-Aldrich)/day for 5 consecutive days. As a control, corn oil was used. All mice used in this study were housed under standard laboratory conditions on a 12 h light/dark cycle and received food and water *ad libitum*.

### BaCl_2_ injection for muscle injury and regeneration analysis

BaCl_2_ (1.2%) was dissolved in PBS and the solution vortexed extensively and sterilized with a 0.22 μm filter. Mice were anesthetized with an intraperitoneal injection of ketamine (Vetone) and zylazine (DailyMed) mixture (100 mg-10 mg/kg) just before BaCl_2_ treatment. BaCl_2_ solution was injected into the left tibialis anterior (TA) muscle by a disposable syringe with a 31-gauge ultrafine needle (#324907, Becton Dickinson). The same volume of sterilized PBS was injected into the TA of control mice.

### Mouse behavior physical tests

Body composition of each mouse (fat and lean mass) was analyzed before and after test IGT. Inverted grip test was performed at the NHLBI Murine Phenotyping Core facility before and after BaCl_2_ injection in the hind leg TA muscle. Briefly, body composition analysis was performed using Bruker Mini-Spec Body Composition Analyzer or EchoMRI NMR machine to non-invasively measure fat, free body fluid and lean tissue values in mice at periodic intervals. Un-anesthetized mice were placed in a clear plastic tube and gently restrained at the end of the tube by using a plunger with air holes. The plunger was fitted to the mouse and tightened just sufficiently to minimize movement. The tube was then inserted into the mini-spec port to a premeasured depth and the measurements were collected. The tube was sanitized by washing with a mild detergent and drying after each use.

Inverted grid was performed using a metal wire grid IGT. Mice were placed in the center on top of a metal wire grid. The grid was slowly inverted 180 degrees approximately two feet above a padded surface. The time the mouse could hang on the grid was recorded. Mice received three trials, and the maximum trial time was 90 s and they received at least 10 min of rest between trials.

### Primary culture of individual myofibers and their satellite cells

Single muscle fibers isolation and primary satellite cells culture were performed according to a previous report [57]. Briefly, extensor digitorum longus (EDL) muscles of mice were incubated in DMEM (Dulbecco’s modified Eagle’s medium; high glucose, L-glutamine with 110 mg/ml sodium pyruvate, #11995073, ThermoFisher Scientific) with 0.2% collagenase type I (#SCR103, Sigma-Aldrich) for approximately 60 min at 37 °C. Single myofibers, mechanically isolated by gentle pipetting, were then cultured in growth medium (GM) for satellite cells (DMEM with 20% fetal bovine serum (FBS), 1% Chicken Embryo Extract (CEE, #C3999, USBiological), 1% penicillin-streptomycin, and fibroblast growth factor-2 (FGF-basic, 25 ng/ml,#450 − 33, Peprotech)) on Matrigel (#356234, Corning)-coated dishes for 6–7 days. Migrated satellite cells from the single fiber were then expanded by subculture and used for further analysis.

### Histology, fiber number, and fiber size analysis

Histology of mouse leg muscle samples was performed according to a previous report [58]. Briefly, samples embedded in tragacanth gum (Sigma) was snap-frozen in liquid nitrogen cooled isopentane (methylbutane, Sigma) and stored at −80 °C until use. Specimens were sectioned at 7 μm with a Cryostat and dried before histological hematoxylin & eosin (H&E) staining or immunofluorescent staining. After fluorescence staining, fiber numbers and diameters was analyzed with Photoshop CC 2015 and fiber CSA area was calculated using Fiji [59].

### In situ hybridization

Whole mount in situ hybridization was performed according to the previous report. Mouse *Scgb3a1* cDNA (that is corresponding to variant 2: NM_170727.2) was cloned by RT-PCR using total RNA from E11.0 mouse embryo as a template and inserted into pGEM-T easy vector (#A1360, Promega). Following primers were used for mouse *Scgb3a1* cDNA cloning: (Forward) 5’-CCCACTAGTCTGATGACATCTTC-3’, (Reverse) 5’-ATCTCGTCAGGCATCTCGTC-3’.

### Immunostaining

For immunostaining of muscle single fibers, mechanically dissociated myofibers as described above were washed with PBS three times, and immediately fixed with 10% formalin for 10 min, and used for immunostaining. After treatment with cold MeOH at −20 °C for 10 min to increase permeability, cells were blocked with 5% BSA for 1 h, followed by staining with primary antibodies described within each Fig. and listed in Supplementary Table S1 for 1 h at room temperature (RT). Alexa flour 488/647/594 antibodies (Invitrogen) were used as a secondary antibodies and nucleus was counter stained with 4’,6-diamidino-2-phenylindole (DAPI) for 10 min at RT. After extensive washing, samples were analyzed under fluorescent microscope BZ-X700 (Keyence) or confocal microscope LSM 780 (Zeiss) according to the instruction of the CCR microscopy core.

### C2C12 cell culture

C2C12 mouse myoblast cell line was purchased from the American Type Culture Collection (#CRL-1772, ATCC). Cells were seeded on Collagen I (#CB-40236, Corning)-coated 6 well plates (Corning) in growth medium (GM: DMEM with 10% FBS) at 37 °C, 5% CO2 in humidified chamber. To induce muscle differentiation, the medium was changed to differentiation medium (DM: DMEM with 2% horse serum (HS, #30–2040, ATCC)) and cells were further incubated. For SCGB3A1 treatment, recombinant mouse HIN-1/SCGB3A1 (#2954-HN-050, R&D Systems) was reconstituted with 1% bovine serum albumin (BSA) in PBS and added into culture medium as described in Figs.

### Quantitative RT-PCR (qPCR)

Total RNA was extracted by TRIzol^®^ (Life Technologies) and reverse transcribed into cDNA by using SuperScript III reverse transcriptase (Life Technologies) according to the manufacturer’s protocol. Analysis of mRNA levels was performed on a 7900 Fast Real-Time PCR System (Life technologies) with SYBR Green-based real-time PCR. Expression levels were normalized with *Gapdh.* Primers used in this manuscript were as follows (See also Supplementary Table S2).

*Myh1* (5’- CCAGGACCTTGTGGACAAAC-3’, 5’- ATTTGGCCAGGTTGACATTG-3’),

*Myh4* (5’-GCAGGACTTGGTGGACAAAC − 3’, 5’-ACTTGGCCAGGTTGACATTG − 3’),

*Notch 3* (5’-CAATGCAGTGGATGAGCTTG − 3’, 5’-GGCTCCATTTTTCAGCAGAG-3’),

*Ckm* (5’- AGGAGATTCTCACTCGCCTTC-3’, 5’- GTTGGAGATGTCGAACACG-3’),

*Gapdh* (5’-CCTTCCGTGTTCCTACCCC-3’, 5’-CCTGCTTCACCACCTTCTTG-3’),

*Hes1* (5’- CTACCCCAGCCAGTGTCAAC-3’, 5’-CGCCTCTTCTCCATGATAGG-3’),

*Hey1* (5’-CACCTGAAAATGCTGCACAC-3’, 5’-ACCCCAAACTCCGATAGTCC-3’), and

*HeyL* (5’-TTTCTGAATTGCGACGATTG-3’, 5’-ACGGTCATCTGCAAGACCTC-3’).

### *Scgb3a1* gene overexpression using retrovirus system

A pMCs-puro-retroviral vector [60] containing mouse *Scgb3a1* whole ORF (variant 1(NM_054037.2) or variant 2 (NM_170727.2)) was transfected into Phoenix packaging cells by using X-treme Gene™ HP DNA transfection reagent (Roche Applied Science). Collected supernatant was added into C2C12 cells culture media and allowed for further incubation. Drug selection and cell cloning were conducted in the presence of 2 µg/ml puromycin by the limited dilution method.

### Western blotting

Mouse tissues or cultured cells were dissolved in RIPA buffer containing phenylmethylsulfonyl fluoride (PMSF) and stored at −80 °C until use. Protein concentrations were measured by use of the Pierce BCA protein assay kit (#23225, ThermoFisher Scientific) as bovine serum albumin as standard. Ten mg of each protein was dissolved in SDS sample buffer and after heated 5 min at 95 °C, samples were loaded onto 4–20% Criterion Tris-HCL precast gel (#3450104, Bio Rad). After electrophoresis and protein separation, proteins were transferred onto PVDF membrane (GE Healthcare). The membrane was probed with primary antibody then with HRP conjugated secondary antibodies (Invitrogen), and the signal was developed using Supersignal West dura (#34075, ThermoFisher Scientific). Chemiluminescence was quantitated using a Bio-Rad ChemiDoc™ MP imaging system.

### Statistical analysis

Statistical analysis was performed using an unpaired two-tailed Student’s *t*-test for the two samples comparison, using Grapfpad Prism 7.0. For all experiments, the data set from triplicate samples were calculated and expressed as means ± standard deviation (SD) or standard error of the mean (SEM). The experiments were repeated three times and representative data were used. *P* < 0.05 were considered statistically significant.

## Supplementary Information

Below is the link to the electronic supplementary material.

Supplementary File 1 (PDF 11.6 MB)

## Data Availability

All data needed to evaluate the conclusions in the paper are present in the paper and/or the Supplementary Materials. Further information and requests for resources and reagents should be directed to and will be fulfilled by the lead contact Shioko Kimura (kimuras@mail.nih.gov). *Scgb3a1*^*−/−*^, *Scgb3a1*^*f/f*^, *Scgb3a1*^*GFP*^ and *Scgb3a1*^*CreERT2*^ knock-in mice will be available upon request from the lead contact with a completed Materials Transfer Agreement.
