# Supplementary material for: Secretoglobin 3A1 in activated muscle satellite cells contributes to myosin heavy chain IIX and IIB fiber differentiation
Source: Cell Mol Life Sci. 2025 Dec 31;83(1):52. doi: 10.1007/s00018-025-06045-5 (PMC12804533; doi:10.1007/s00018-025-06045-5)
Supplement: Supplementary file 1 — (PDF 11.6 MB) [file 18_2025_6045_MOESM1_ESM.pdf]

Supplementary materials

Secretoglobin 3A1 in activated muscle satellite cells contributes to myosin heavy chain IIX and IIB fiber differentiation

*Shigetoshi Yokoyama, Taketomo Kido, Mitsuhiro Yoneda, Danielle A. Springer, Parirokh P. Awasthi, Raj Chari, Andrew D. Patterson, and Shioko Kimura**

This file includes:

Figs. S1 to S11
Table S1, S2

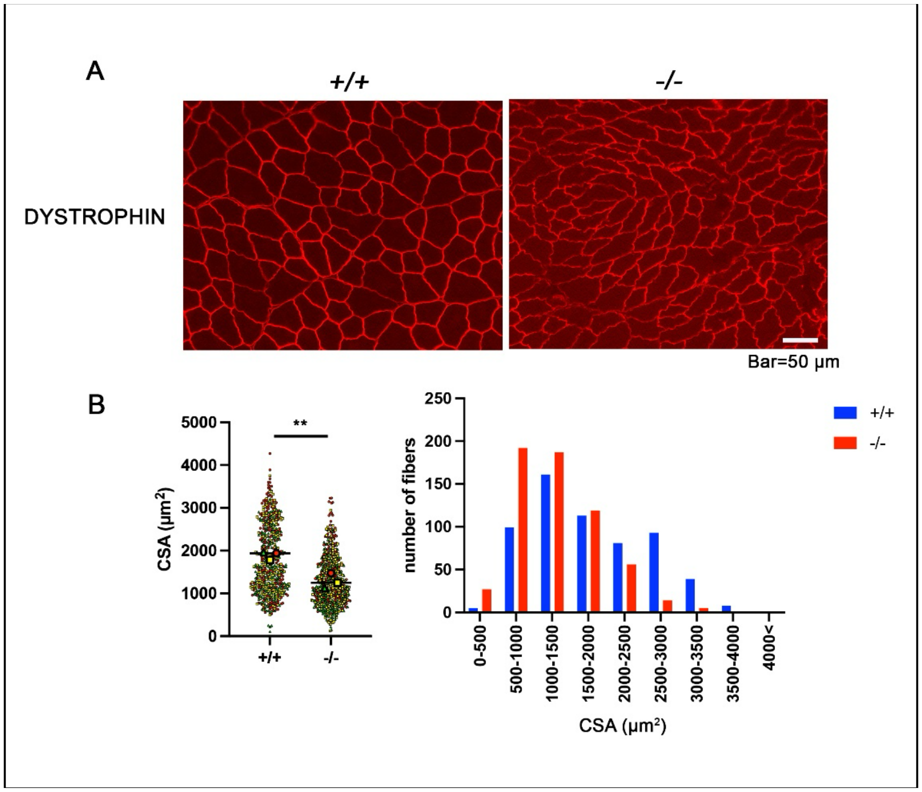

Fig. S1 Immunostaining of DYSTROPHIN in 18-week-old *Scgb3a1*$^{+/+}$ and *Scgb3a1*$^{-/-}$ TA muscle.
(**A**) Representative images of immunofluorescent staining of TA muscle of 18-week-old *Scgb3a1*$^{+/+}$ (+/+) or *Scgb3a1*$^{-/-}$ (-/-) mice for the DYSTROPHIN expressions. Bar= 50 µm.
(**B**) Details of muscle fiber size distribution between +/+ and -/- mice. Right Figure depicts the distribution of fiber size (n=3 mice, each 100 fibers per mouse). ***p*<0.01 by Student's *t*-test.

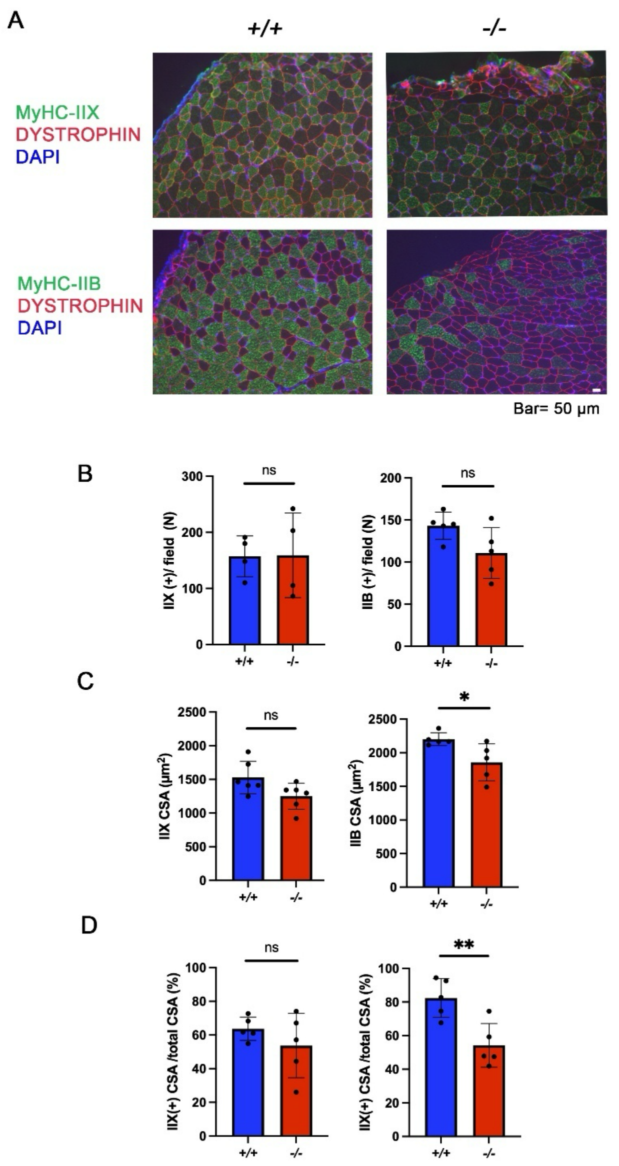

Fig. S2 Immunostaining of MyHC-IIX and MyHC-IIB in 4-week-old *Scgb3a1*$^{+/+}$ and *Scgb3a1*$^{-/-}$ TA muscle.

(**A**) Representative images of immunofluorescent double staining of TA muscle of 10-month-old *Scgb3a1*$^{+/+}$ (+/+) or *Scgb3a1*$^{-/-}$ (-/-) mice for the MyHC-IIX and DYSTROPHIN or MyHC-IIB and DYSTROPHIN expressions in intact TA muscle. Bar= 50 µm. (n=3). (**B**) Each MyHC fiber numbers per independent field (n=5). (**C**) Average CSA of MyHC-IIX and -IIB between +/+ and -/- mice (n=5 independent fields). (**D**) Average each MyHC (+) CSA per total muscle CSA between +/+ and -/- mice (n=5 independent fields). Total fiber number; IIX>600, IIB>500. *p<0.05, **p<0.01 by Student's t-test. ns, not significant.

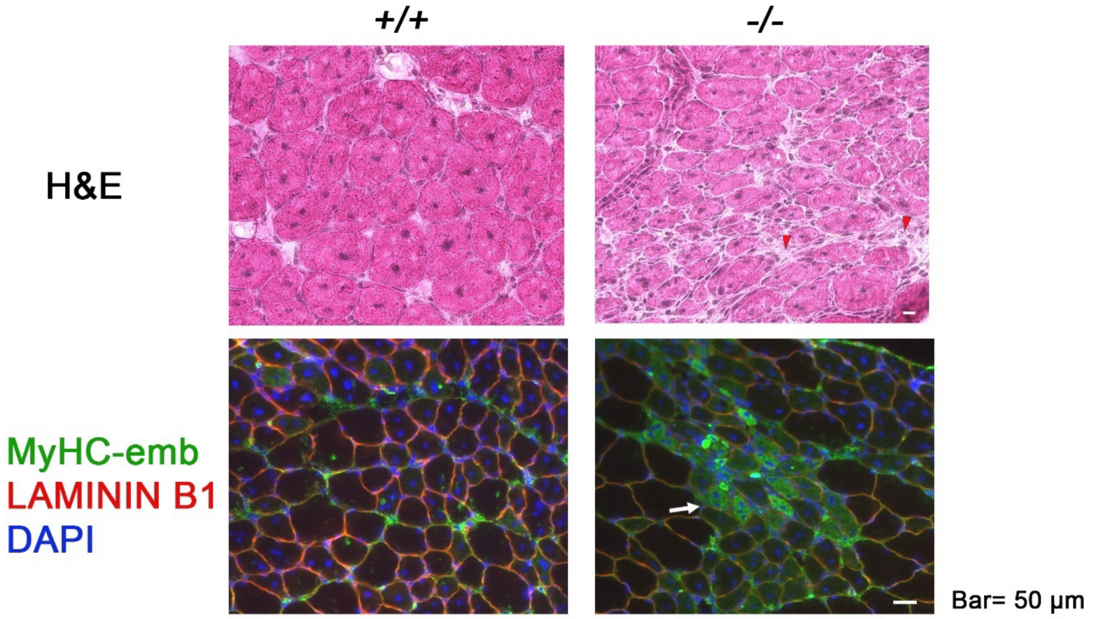

Fig. S3 TA muscle of *Scgb3a1*$^{+/+}$ and *Scgb3a1*$^{-/-}$ 1 week after $BaCl_2$ injection.
(**Upper**) Representative image of HE staining of TA muscle after 1 week of $BaCl_2$ injection. Red arrowheads indicate large interstitial region. (**Bottom**) Immunostaining of Embryonic MyHC (MyHC-emb) and LAMININ B1 after 1 week of $BaCl_2$ injection. White arrow shows expression of MyHC-emb. Bar=50 μm. Nuclei were counterstained with DAPI.

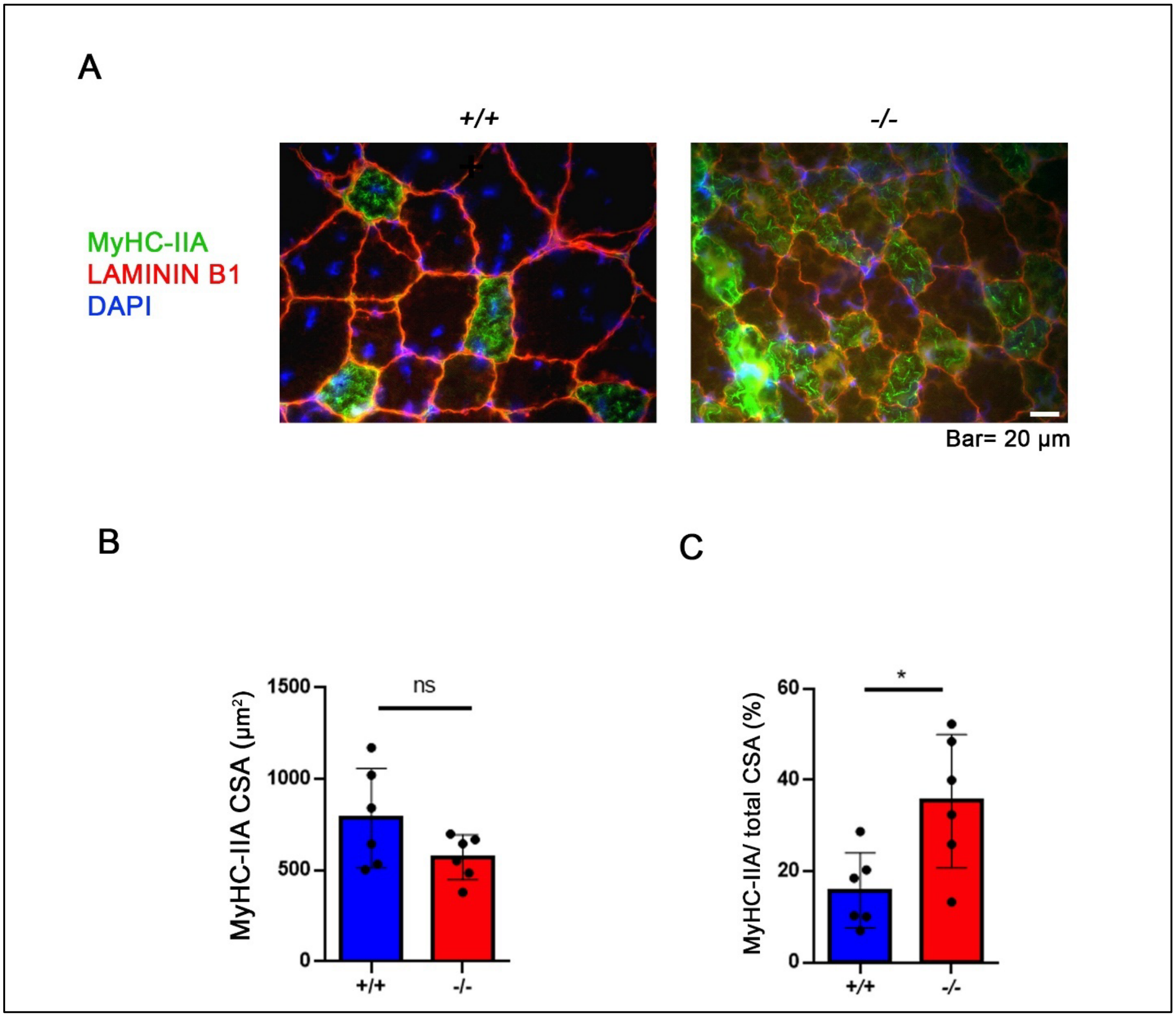

Fig. S4 MyHC IIA immunostaining in *Scgb3a1*$^{+/+}$ and *Scgb3a1*$^{-/-}$ tissues after $BaCl_2$ injection. (**A**) Representative images of immunofluorescent double staining of TA muscle of 1-month-old *Scgb3a1*$^{+/+}$ (+/+) or *Scgb3a1*$^{-/-}$ (-/-) mice for the MyHC-IIA and LAMININ B1 expressions after $BaCl_2$ injection. Bar= 20 µm. (n=3). (**B**) MyHC-IIA (+) fiber size CSA between *Scgb3a1*$^{+/+}$ (+/+) or *Scgb3a1*$^{-/-}$ (-/-) mice after $BaCl_2$ injection (n=6 independent fields/3 mice). (**C**) Percentage of MyHC-IIA+ area per total muscle area between *Scgb3a1*$^{+/+}$ (+/+) or *Scgb3a1*$^{-/-}$ (-/-) mice after $BaCl_2$ injection (n=6 independent fields/3 mice). Total IIA fibers>200. *$p<0.05$ by Student's *t*-test. ns, not significant.

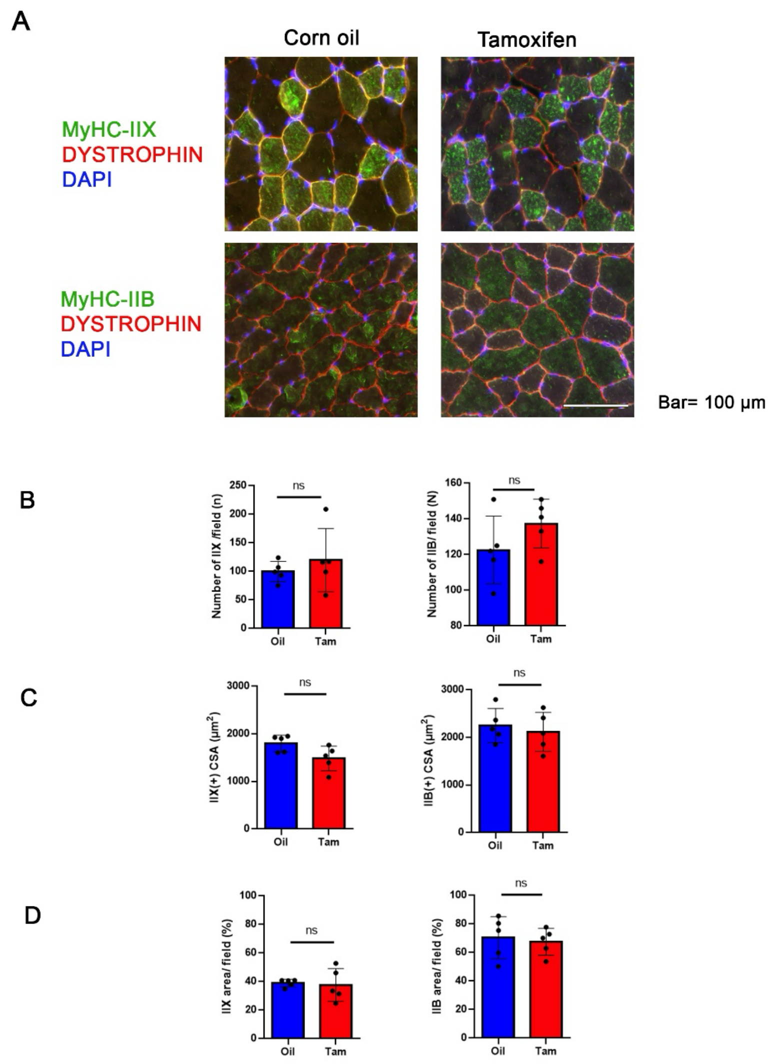

Fig. S5 Immunofluorescent images of MyHC-IIX and MyHC-IIB in $Pax7^{CreERT2}$;$Scgb3a1^{f/f}$ mice treated with or without tamoxifen.

(**A**) Representative image of MyHC-IIX or MyHC-IIB and DYSTROPHIN in intact TA muscle of 4 weeks after administration of corn oil (Control) or Tamoxifen (CKO). (**B**) Each MyHC fiber numbers per independent field (n=5). (**C**) Average CSA of MyHC-IIX and -IIB between +/+ and -/- mice (n=5 independent fields). (**D**) Average each MyHC (+) CSA per total muscle CSA between +/+ and -/- mice (n=5 independent fields). Total fiber number>500. ns, not significant by Student's *t*-test.

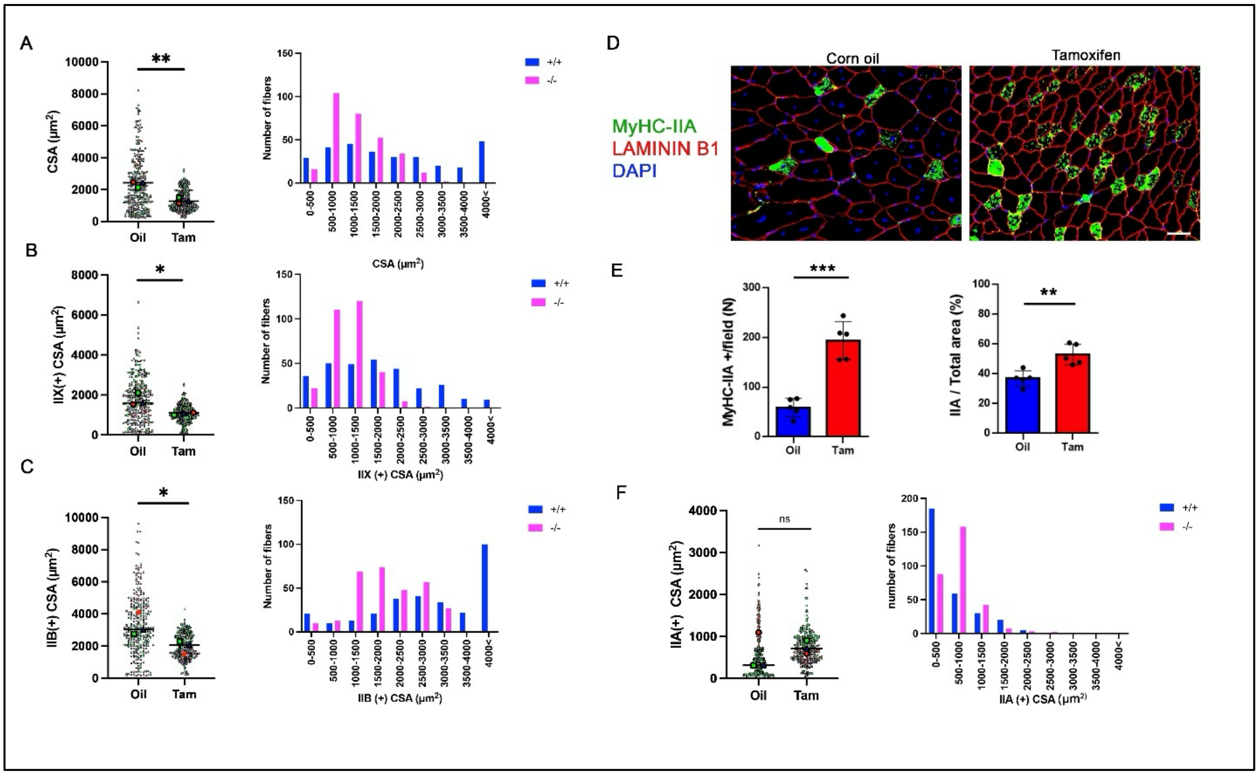

Fig. S6 *Pax7*^{CreERT2};*Scgb3a1*$^{f/f}$ mice treated with Tam show severe MyHC-IIX, IIB differentiation defects.

(**A**) Distribution of CSA of HE stained TA muscle of Oil (control) or Tam treated followed by BaCl$_2$ injection. n=3 mice, each 100 fibers per mouse. (**B**) Distribution of CSA of MyHC-IIX (+) fibers determined by immunofluorescent analysis. n=3 mice, each 100 fibers per mouse. (**C**) Distribution of CSA of MyHC-IIB (+) fibers determined by immunofluorescent analysis. n=3mice, each 100 fibers per mouse. (**D**) Double immunostaining of MyHC-IIA and LAMININ B1 of control or Tamoxifen treated TA muscle followed by BaCl2 injection. Nuclei were counterstained with DAPI. Bar=50 µm. (**E**) (Left) MyHC-IIA+ fiber numbers per fields (n=5, total fiber numbers>1000). (Right) Percentage of MyHC-IIA area per total area using data shown on left graph (n=5 independent fields). (**F**) Distribution of CSA of MyHC-IIA (+) fibers determined by immunofluorescent analysis. n=3 mice, each 100 fibers per mouse. *p<0.05, **p<0.01, ***p<0.005, ns; not significant by Student's *t*-test.

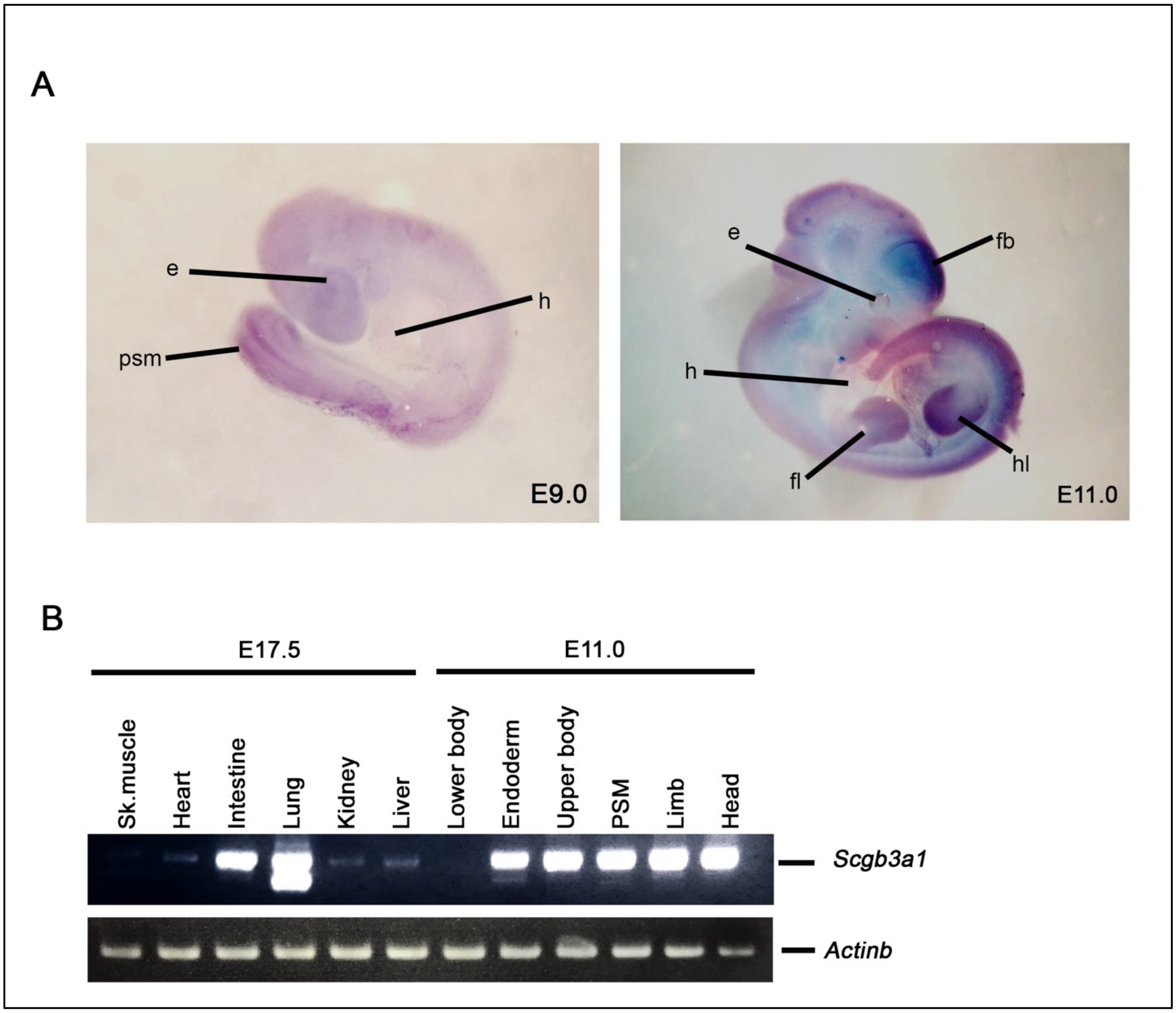

Fig. S7 Expression of *Scgb3a1* mRNA in mouse embryos.
(**A**) Whole-mount in situ hybridization was carried out using mouse E9.0 (left) or E11.0 (right) embryos with DIG-*Scgb3a1* antisense RNA probe. n=3. e, eye; h, heart; psm, presomitic mesoderm; fb, forebrain; fl, forelimb; hl, hindlimb. (**B**) PCR results for *Scgb3a1* mRNA in E17.5 and E11.0 tissues. E11.0 endoderm is a mixture of heart and liver. PSM, presomitic mesoderm.

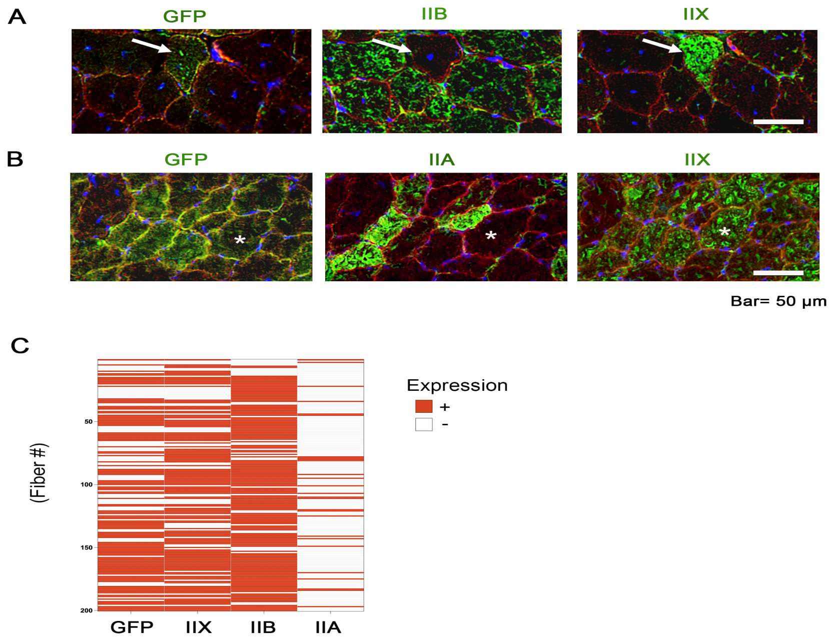

	Total	IIX	IIB	IIA
All fibers	200	161 (80.5)	116(58.0)	80(40.0)
GFP+ fibers	121	112(92.6)	100(82.6)	13(10.7)

Fig. S8 GFP (+) fibers are composed of MyHC-IIX (+) fibers.
(**A**) Double immunofluorescent staining of GFP (green) and tdTomato (red) (left), MyHC-IIB (green) and DYSTROPHIN (red) (center), and MyHC-IIX (green) and DYSTROPHIN (red) (right) in serial sections of TA muscle of *SCGB3A1*^{CreERT2}*;mT/mG* knock-in mice after 3 weeks of $BaCl_2$ injection. Nuclei were counterstained with DAPI (blue). Arrows depict the same fiber. Bar= 50 µm. (**B**) Double immunofluorescent staining of GFP and tdTomato (left), MyHC-IIA and DYSTROPHIN (center), and MyHC-IIX and DYSTROPHIN (right) in serial sections of TA muscle of *SCGB3A1*^{CreERT2}*;mT/mG* knock-in mice after 3 weeks of $BaCl_2$ injection. Nuclei were counterstained with DAPI (blue). Asterisks depict the same fiber. Bar= 50 µm.
(**C**) Quantification of co-expression of GFP with MyHC-IIX, MyHC-IIB, and MyHC-IIA in 200 fibers. Red bars indicate positive expressions in each fiber. Bottom: Total number of fibers of each fast-type MyHC coinciding with GFP expression or with GFP+ fibers. Numbers in parentheses indicate the percentage relative to the GFP expression pattern or to GFP+ fibers.

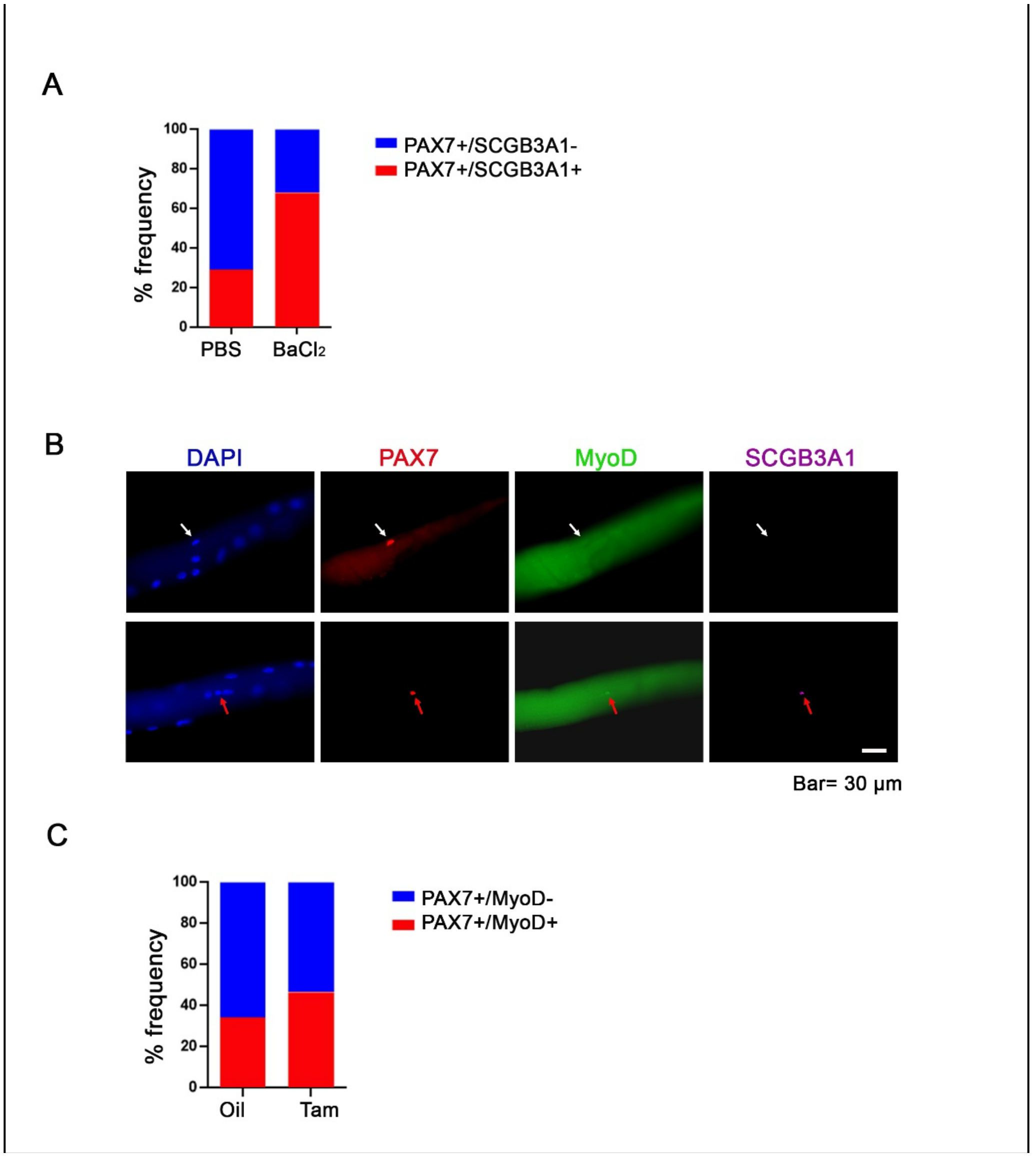

Fig. S9 SCGB3A1 is colocalized with PAX7+/MyoD+ activated satellite cells.
(**A**) Percentage of PAX7+/SCGB3A1- or PAX7+/SCGB3A1+ satellite cells in control (PBS) or $BaCl_2$ treated EDL muscle examined 1 week after $BaCl_2$ treatment. n=3 mice. Total number of PAX7(+) cells= PBS; 17, $BaCl_2$; 50. (**B**) Immunostaining of EDL muscle with $BaCl_2$ injection carried out at 4 weeks of age. Muscles were collected 1 week after $BaCl_2$ injection. Individually separated single muscle fibers were stained with anti-PAX7, anti-MyoD and anti-SCGB3A1 antibodies. Representative image of PAX7(+)/MyoD(-)/SCGB3A1(-) (white Arrows) and PAX7(+)/MyoD(+)/SCGB3A1(+) cells (red arrows) after $BaCl_2$ injection. Nuclei were counterstained with DAPI. Bar=30 μm. (**C**) Percentage of quiescent (PAX7+/MyoD-) or activated (PAX7+/MyoD+) satellite cells in control (oil) or CKO (Tam) EDL muscle of 26-week-old mice. n=3 mice. Total number of PAX7(+) cells = Oil; 61, Tam; 86.

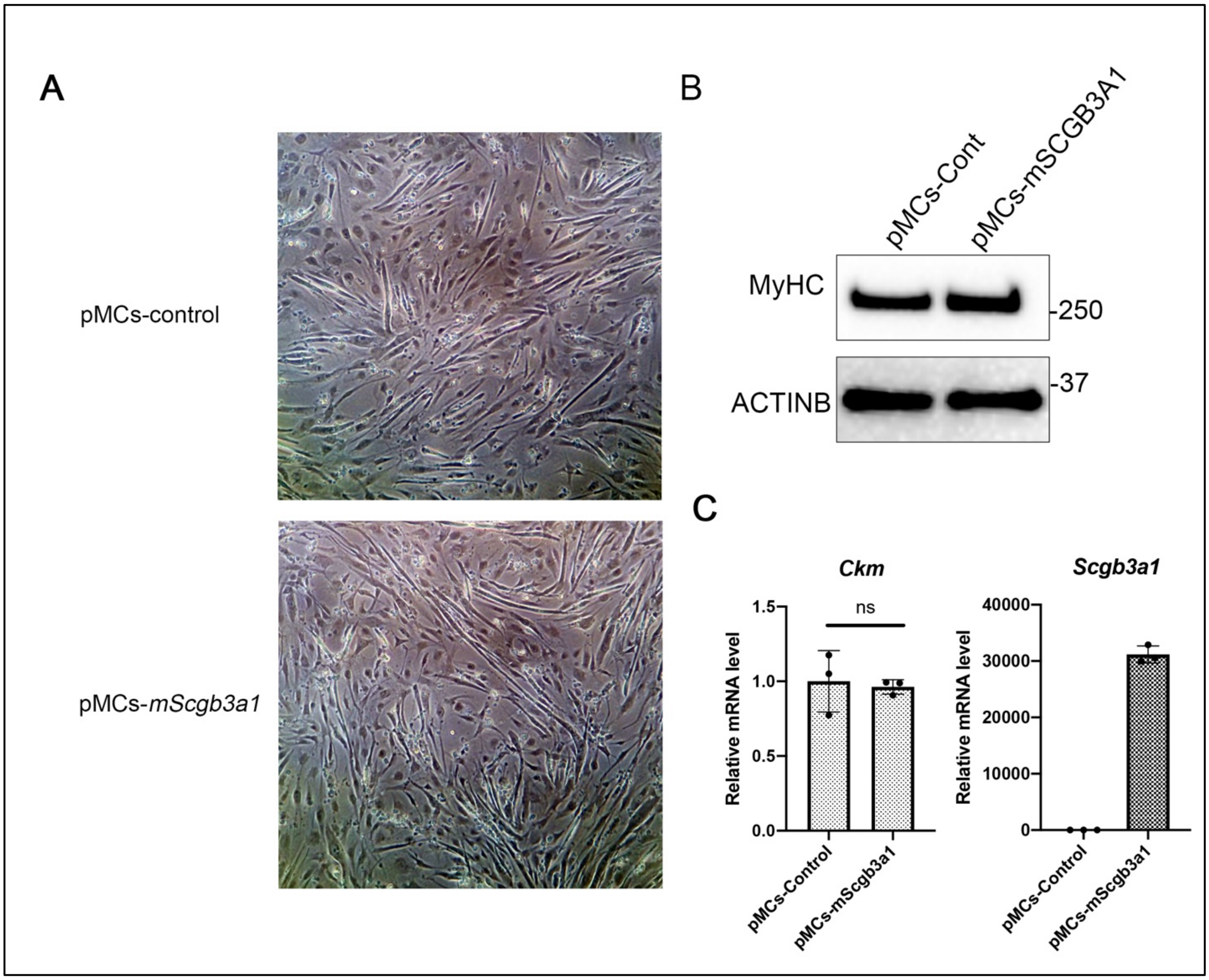

Fig. S10 Constitutive *Scgb3a1* expression in C2C12 does not promote terminal differentiation. (**A**) Overall morphology of cell culture of C2C12-pMCs-*mScgb3a1* or equivariant control vector clone for 7 days in DM (differentiation medium). (**B**) Immunoblotting results of culture of (A) for anti-MyHC antibody. Anti-ACTINB antibody was used for internal loading control. (**C**) qPCR results of culture of (A) for *Creatin muscle kinase* (*Ckm*) and *Scgb3a1*. All data were normalized to *Gapdh* gene. n=3. qPCR was carried out in triplicate per sample. ns: not significant by Student's *t*-test.

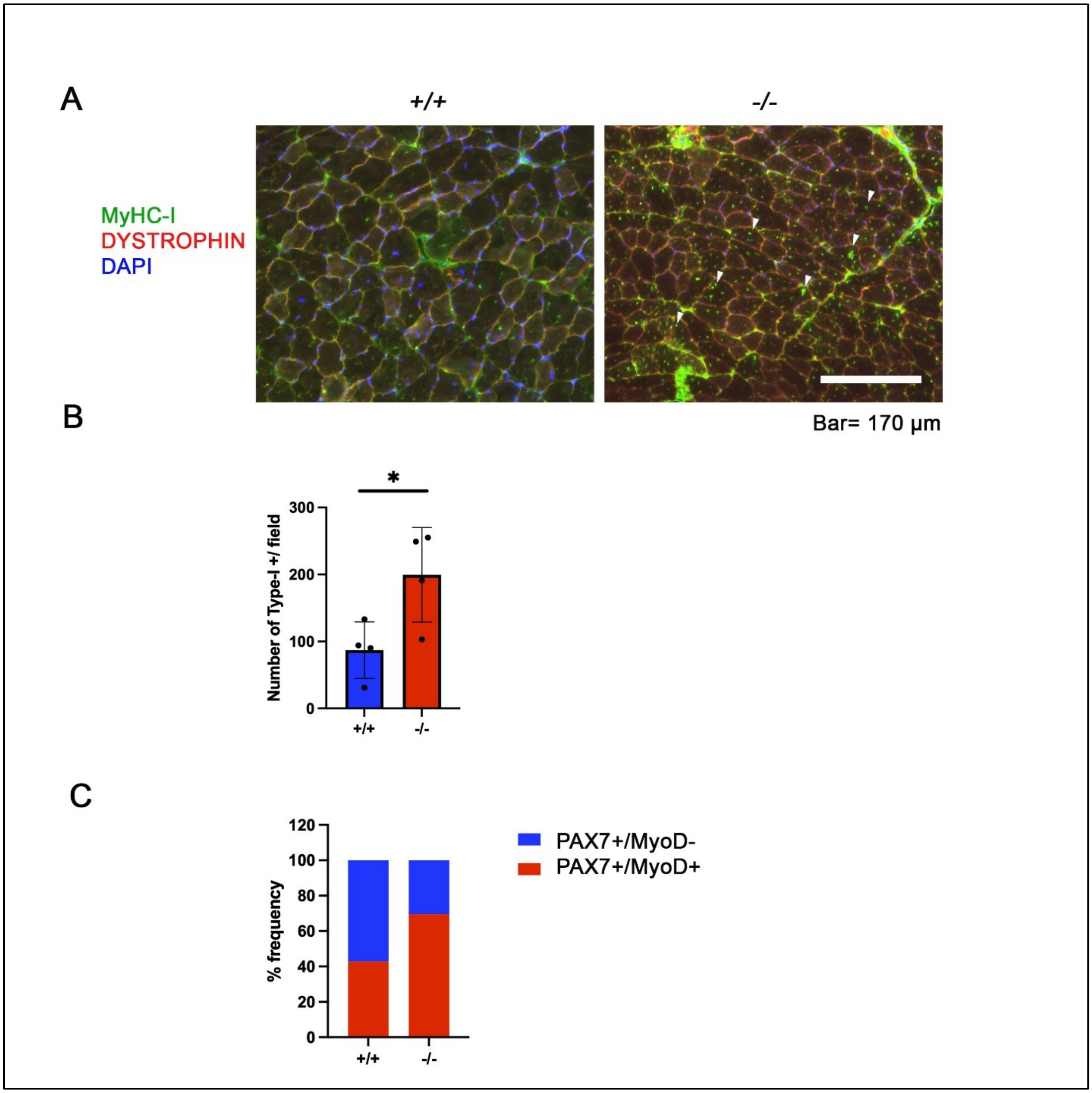

Fig. S11 Aged *Scgb3a1*-/- mice exhibit MyHC-I in intact TA muscles.
(**A**) Representative images of immunofluorescent double staining of TA muscle of 22-month-old Scgb3a1$^{+/+}$ (+/+) or Scgb3a1$^{-/-}$ (-/-) mice for the MyHC-I and DYSTROPHIN expressions. Arrowheads indicate the emergence of scattered MyHC-I expressions in *Scgb3a1*-/- TA muscles. Bar= 170 µm. (n=3). Nuclei were counter stained with DAPI. (**B**) MyHC-I+ fiber numbers per fields (n=4, total fiber numbers>500). (**C**) Percentage of quiescent (PAX7+/MyoD-) or activated (PAX7+/MyoD+) satellite cells in aged Scgb3a1$^{+/+}$ (+/+) or Scgb3a1$^{-/-}$ (-/-) EDL muscle of 84−week-old mice. n=3 mice. *p<0.05 by Student's *t*-test.

Table S1

REAGENT or RESOURCE	SOURCE	IDENTIFIER
Antibodies		
Mouse anti-Myosin heave chain (MyHC) (clone MF20)	DSHB	#MF20, RRID:AB_2147781
mouse anti-MyHC Type I (BA-D5)	DSHB	#BA-D5, RRID:AB_2235587
mouse anti-MyHC IIA (sc-71)	DSHB	#sc-71, RRID:AB_2147165
mouse anti-MyHC IIX (6H1)	DSHB	#6H1, RRID:AB_1157897
mouse anti-MyHC IIB (BF-F3)	DSHB	#BF-F3, RRID:AB_2266724
mouse anti-MyHC-neonatal (N3.36)	DSHB	#N3.36,RRID:AB_528380
mouse anti-Pax7 (DSHB)	DSHB	#PAX7, RRID:AB_2299243
rat anti-HIN1/SCGB3A1	R&D	#MAB2954, RRID:AB_2157009
goat anti-HIN1/SCGB3A1	R&D	#AF2954, RRID:AB_2157011
rabbit anti-Notch3	Proteintech	#55114-1-AP, RRID:AB_10858393
rabbit anti-MyoD	Santa Cruz Biotechnology	#sc-760, RRID:AB_2148870
rat anti-Laminin beta-1 (LT3)	Thermo Fisher Scientific	#MA5-14567, RRID:AB_10981503
mouse anti-Gapdh	Proteintech	#60004-1-Ig, RRID:AB_2107436
rabbit anti-Caspase 3	Cell Signaling Technology	#D3R6Y, RRID:AB_2798429
rabbit anti-Dystrophin	Thermo Fisher Scientific	#PA1-21011, RRID:AB_2091358
rabbit anti-Ki67	Abcam	#ab16667, RRID:AB_302459
mouse anti-Actin (a-Sarcomeric) clone 5C5	SIGMA	#A2172, RRID:AB_476695
rabbit anti-b-actin	Cell Signaling Technology	(#4967, RRID:AB_330288
Bacterial and virus strains		
Biological samples		
Chemicals, peptides, and recombinant proteins		
Tamoxifen		
BaCl2	Sigma	# 202738
PBS	Fisher Scientific	#10-010-072

DMEM (Dulbecco's modified Eagle's medium; high glucose, L-glutamine with 110 mg/ml sodium pyruvate	ThermoFisher Scientific	#11995073
collagenase type I	SIGMA	#SCR103
FBS		
Chicken Embryo Extract	USBiological	#C3999
penicillin - streptomycin	Gibco	#15140122
fibroblast growth factor-2 (FGF-basic)	Peprotech	#450-33
Collagen I	Corning	#CB-40236
horse serum (HS)	ATCC	#30-2040
recombinant mouse HIN-1/SCGB3A1	R&D Systems	#2954-HN-050
Formalin		
MeOH	SIGMA	# 1424109
bovine serum albumin (BSA)	Caibiochem	#2960
4',6-diamidino-2-phenylindole (DAPI)		
tragacanth gum	SIGMA	# G1128
isopentane (methylbutane)	SIGMA	#277258
RIPA buffer	ThermoFisher Scientific	#89900
PMSF	SIGMA	#41216525
4-20 % Criterion Tris HCL precast gel	Bio Rad	#3450104
PVDF	GE Healthcare	# RPN303F
Supersignal West dura	ThermoFisher Scientific	#34075
TRIzol®	Life Technologies	#15596026
SuperScript III reverse transcriptase	ThermoFisher Scientific	# 18080044
puromycin		
Critical commercial assays		
Pierce BCA protein assay kit	ThermoFisher Scientific	#23225
X-treme Gene™ HP DNA transfection reagent	Roche	#6366244001
Deposited data		
Experimental models: Cell lines		
C2C12	ATCC	N/A

Experimental models: Organisms/strains		
Mouse:Scgb3a1-KO	This study	
Mouse:Pax7-CreERT2	The Jackson Laboratory	
Mouse: Scgb3a1 f/f	This study	
Oligonucleotides		
Primers for qRT-PCR, genomic PCR, see Table S1.	This study	
Recombinant DNA		
pGEM-T-mScgb3a1	This study	
pMCs-mScgb3a1-v1	This study	
pMCs-mScgb3a1-v2	This study	
Software and algorithms		
GraphPad Prism (version 7)	GraphPad Software,	https://www.graphpad.com
Zen Black (version 3.1)	Zeiss	https://www.zeiss.com/microscopy/int/products/microscope-software/zen-lite
Adobe Photoshop CC	Adobe	https://www.adobe.com/uk/products/photoshop
imageJ	imageJ	https://imagej.nih.gov/ij/index.html
Other		

Table S2. List of primers used in this study, related to Methods

Primers used in this study:

Amplicon	F primer (5'-->3')	R primer (5'-->3')	Used for	Reference
Scgb3a1 KO	GGTGTTGCTTTCTTCATGGAC	AGCTCGGTGACACACTTCCT	detection KO allele	This study
Scgb3a1 EX2	TGGCCACCTTTCCTAACTTC	AAGGGGAAACTGGGTGACAT	wild-type Scgb3a1 exon2	This study
mScgb3a1	CTGAGGCTGTAGGAGCTGTG	ATCTCGTCAGGCATCTCGTC	qPCR	This study
m*Myh1*	CCAGGACCTTGTGGACAAAC	ATTTGGCCAGGTTGACATTG	qPCR	This study
m*Myh4*	GCAGGACTTGGTGGACAAAC	ACTTGGCCAGGTTGACATTG	qPCR	This study
m*Notch3*	CAATGCAGTGGATGAGCTTG	GGCTCCATTTTTCAGCAGAG	qPCR	This study
m*Hes1*	CTACCCCAGCCAGTGTCAAC	CGCCTCTTCTCCATGATAGG	qPCR	This study
m*Hey1*	CACCTGAAAATGCTGCACAC	ACCCCAAACTCCGATAGTCC	qPCR	This study
m*HeyL*	TTTCTGAATTGCGACGATTG	ACGGTCATCTGCAAGACCTC	qPCR	This study
m*Gapdh*	CCTTCCGTGTTCCTACCCC	CCTGCTTCACCACCTTCTTG	qPCR	This study
m*Ckm*	AGGAGATTCTCACTCGCCTTC	GTTGGAGATGTCGAACACG	qPCR	This study
m*Scgb3a1*-cDNA	CCCACTAGTCTGATGACATCTTC	ATCTCGTCAGGCATCTCGTC	ISH probe	This study
Scgb3a1-P2ACreERT2	CAGGTGTTGCTTTCTTCATGG	GTTAACCTCAAGCTGTGGCAG	detection P2ACreERT2 allele	This study
Scgb3a1-GFP	CAGGTGTTGCTTTCTTCATGG	AGACAATCTCCAGAGTCTGG	detection GFP allele	This study

Primers used for preparation of *Scgb3a1* transgenic mice:

Candidate guide RNA	Target site (PAM in bold)
1533	CAATCCTCGGTAGCAGTGAA**GGG**
1534	AGTGCCCTTCACTGCTACCG**AGG**
1535	GTCCTGACAATGTTCGGTTG**AGG**
1536	TCCTGACAATGTTCGGTTGA**GGG**
1537*	CCTGACAATGTTCGGTTGAG**GGG**
1538	ATCTCGTCAGGCATCTCGTC**AGG**
**To generate ssDNA**	
Scgb3a1-ssDNA-F	CCTAGCCTACCATTAAGCCA
Scgb3a1-ssDNA-R	/5Phos/CAAACAGGTCATCTATATGTGTT

*Candidate guide RNA used for mouse generation
PAM: protospacer adjacent motif
